# An Antigen-Presenting and Apoptosis-Inducing Polymer Microparticle Prolongs Alloskin Graft Survival by Selectively and Markedly Depleting Alloreactive CD8^+^ T Cells

**DOI:** 10.3389/fimmu.2017.00657

**Published:** 2017-06-09

**Authors:** Wei Wang, Khawar Ali Shahzad, Miaochen Li, Aifeng Zhang, Lei Zhang, Tao Xu, Xin Wan, Chuanlai Shen

**Affiliations:** ^1^Department of Microbiology and Immunology, Southeast University Medical School, Nanjing, China; ^2^Department of Pathology, Southeast University Medical School, Nanjing, China

**Keywords:** biomimetic microparticle, poly lactic-co-glycolic acid, peptide–major histocompatibility complex, alloreactive T cells, allograft rejection, killer artificial antigen-presenting cell

## Abstract

Selectively depleting the pathogenic T cells is a fundamental strategy for the treatment of allograft rejection and autoimmune disease since it retains the overall immune function of host. The concept of killer artificial antigen-presenting cells (KaAPCs) has been developed by co-coupling peptide–major histocompatibility complex (pMHC) multimer and anti-Fas monoclonal antibody (mAb) onto the polymeric microparticles (MPs) to induce the apoptosis of antigen-specific T cells. But little information is available about its *in vivo* therapeutic potential and mechanism. In this study, polyethylenimine (PEI)-coated poly lactic-co-glycolic acid microparticle (PLGA MP) was fabricated as a cell-sized scaffold to covalently co-couple H-2K^b^-Ig dimer and anti-Fas mAb for the generation of alloantigen-presenting and apoptosis-inducing MPs. Intravenous infusions of the biodegradable KaAPCs prolonged the alloskin graft survival for 43 days in a single MHC-mismatched murine model, depleted the most of H-2K^b^-alloreactive CD8^+^ T cells in peripheral blood, spleen, and alloskin graft in an antigen-specific manner and anti-Fas-dependent fashion. The cell-sized KaAPCs circulated throughout vasculature into liver, kidney, spleen, lymph nodes, lung, and heart, but few ones into local allograft at early stage, with a retention time up to 36 h *in vivo*. They colocalized with CD8^+^ T cells in secondary lymphoid organs while few ones contacted with CD4^+^ T cells, B cells, macrophage, and dendritic cells, or internalized by phagocytes. Importantly, the KaAPC treatment did not significantly impair the native T cell repertoire or non-pathogenic immune cells, did not obviously suppress the overall immune function of host, and did not lead to visible organ toxicity. Our results strongly document the high potential of PLGA MP-based KaAPCs as a novel antigen-specific immunotherapy for allograft rejection and autoimmune disorder. The *in vivo* mechanism of alloinhibition, tissue distribution, and biosafety were also initially characterized, which will facilitate its translational studies from bench to bedside.

## Introduction

Selectively depleting antigen-specific T cells is one of the fundamental strategies to treat allograft rejection and autoimmune disorders because it prevents intact immune impairment, the major drawback of current immunosuppressive agents (1–3). Killer antigen-presenting cells (KAPCs) thus have been developed by genetically engineered to express the Fas ligand (FasL) onto B cell lines (4), dendritic cells (DCs) (5) or macrophages (6) to induce peripheral antigen-specific apoptosis of T cells (7–14). Although the promising results in the treatment of chronic infections (15, 16), allograft rejection (8, 10, 17), or autoimmune diseases (9) in murine or human models, cell-based FasL-expressing KAPCs still suffer from several significant problems related to their cellular nature: the risk of infection, tumorigenicity or immunogenicity raised by live cells, the time consuming, and cost-intensive generation when scaled, large batch-to-batch variability of FasL expression (18), mature killer DCs may highly express B7.1 or B7.2 and weakly express FasL (19), thereby eliciting vigorous T cell responses toward other antigens and massive neutrophil infiltration (20), and the sensitivity to their *in vivo* and *in vitro* environments due to the activity of cytotoxic T cells, which can lead to KAPC depletion or unwanted changes in cell-cell signaling (21).

To overcome the restrictions associated with cellular KAPCs, attention has shifted toward the acellular killer artificial antigen-presenting cells (KaAPCs), in light of that peptide–major histocompatibility complex (pMHC) multimers can selectively target antigen-specific T cells *in vitro* (22) and *in vivo* (23, 24). In 2008, Schutz et al. developed the first polymeric KaAPCs by covalently coupling pMHC multimer and apoptosis-inducing anti-Fas monoclonal antibody (mAb) onto cell-sized magnetic beads and documented their ability to selectively deplete antigen-specific T cells in static 96-well plates from T-cell populations with diverse antigen specificities in a Fas/FasL-dependent manner (25). Furthermore, their therapeutic potential has been presented by our previous *in vivo* testing. The latex bead-based KaAPCs could selectively deplete 60% alloreactive T cells after two intravenous injections and prolong alloskin survival for 6 days in a full MHC-mismatched murine model, without the loss of overall immune responsiveness (26). However, despite the encouraging results, the use of magnetic or latex beads as an acellular scaffold may evoke concerns regarding biosafety and organ toxicity for the putative clinical use. Therefore, a biodegradable, non-toxic, and biocompatible platform should be further developed.

Poly lactic-co-glycolic acid (PLGA) is a biocompatible and biodegradable polymer approved by the United States Food and Drug Administration (FDA) and has been widely used for delivering small molecule drugs, proteins, and macromolecules in research and clinical settings (27–29). Thus more recently, we generated the antigen-presenting killer PLGA microparticles (MPs) by covalently co-coupling H-2K^b^-Ig dimers and anti-Fas mAbs on the surface of cell-sized and polyethylenimine (PEI)-coated PLGA-MPs. OVA antigen-presenting killer PLGA-MPs could significantly deplete OVA_257–264_-specific CD8^+^ T cells in an antigen-specific manner and Fas/FasL-dependent fashion, both *in vitro* and in OT-1 mice (30). In this study, the promising capability of poly lactic-co-glycolic acid microparticle (PLGA MP)-based KaAPCs to treat alloskin rejection has been validated in a single MHC-mismatched murine model, which can maximally reveal the therapeutic effects of H-2K^b^ alloantigen-targeted KaAPCs for alloskin rejection, without the interference from the alloantigen responses against other mismatched MHC between donor and recipient. More importantly, the *in vivo* mechanisms of alloinhibition, tissue distribution, and the effects of KaAPC administration on diverse immune cells, overall immune function, and organ toxicity in recipient mice have been characterized. These new evidences strongly suggest the potential of this biodegradable KaAPCs as a novel antigen-specific immunotherapy for allograft rejection and autoimmune disease.

## Materials and Methods

### Mice and Cell Lines

The bm1 (B6.C-H2^bm1/ByJ^) mice were purchased from the Jackson Laboratory (Sacramento, CA, USA) and bred in-house. Male C57BL/6J (H-2K^b^) and BALB/c (H-2K^d^) mice were purchased from the Comparative Medicine Center of Yangzhou University (Yangzhou, China). All mice were maintained in the specific pathogen-free Laboratory Animal Centre of Southeast University (Nanjing, China) and used in experiments at 8–10 weeks of age. Animal welfare and experimental procedures were performed in accordance with the National Institutes of Health guide for the care and use of Laboratory animals (NIH Publications No. 8023, revised 1978) and the Guide for the Care and Use of Laboratory Animals (Ministry of Science and Technology of China, 2006) and were approved by the Animal Ethics Committee of Southeast University. B16F10 and Yac-1 cell lines were purchased from the American Type Culture Collection (Manassas, VA, USA).

### Fabrication of PLGA MPs and ICG-Encapsulated PLGA MPs (ICG-MPs)

Poly lactic-co-glycolic acid microparticles and indocyanine green (ICG)-encapsulated PLGA MPs were prepared using a double-emulsion solvent evaporation method and coated by PEI as described in our previous report (30). The resulting MPs were characterized using scanning electron microscopy (SEM, ZEISS EVO 18, Oberkochen, Germany). The size distribution was analyzed using dynamic light scattering (BI-90 Particle Sizer, Brookhaven Instruments Corporation, Holtsville, NY, USA). The zeta potential of MPs was measured using the Zeta PALS instrument (Brookhaven Instruments Corporation).

### Generation of KaAPCs, ICG-Encapsulated KaAPCs, and R-Phycoerythrin (PE)-Labeled KaAPCs

For KaAPCs and ICG-encapsulated KaAPCs generation, PLGA MPs or ICG-MPs (1 × 10^8^) were co-incubated with H-2K^b^-Ig dimer (BD Biosciences, Franklin Lakes, NJ, USA) (10 µg) and anti-Fas mAbs (clone Jo2, BD Biosciences) (10 µg) in sterile 0.1 M PBS at 4°C for 24 h on a rotator and followed by another 24-h incubation in blocking buffer (10% mouse serum in 0.1 M PBS). R-phycoerythrin (PE)-labeled KaAPCs (PE-KaAPCs) were prepared by co-incubating H-2K^b^-Ig (10 µg), anti-Fas mAbs (10 µg), and PE-streptavidin (BD Biosciences) (15 µg) with MPs (1 × 10^8^) in a similar manner. In parallel, anti-Fas-MPs were generated by coupling anti-Fas mAb onto MPs. Blank-MPs were prepared by blocking MPs with bovine serum albumin (BSA).

For phenotype analyses, KaAPCs and control MPs were stained with PE-labeled anti-H-2K^b^ mAbs (clone AF6-88.5, BD Biosciences) and fluorescein-5-isothiocyanate (FITC)-labeled anti-hamster IgG (binds to Fc domain of anti-Fas mAb, clone G192-1, BD Biosciences) for 30 min at 4°C and imaged through confocal laser scanning microscopy (Olympus, Center Valley, PA, USA) and also acquired on a FACSCalibur flow cytometer (BD Biosciences, San Diego, CA, USA). The data were analyzed with FlowJo software (Tree Star, Ashland, OR, USA). Each batch of KaAPCs and control MPs was routinely evaluated in this manner prior to use.

### Skin Transplantation and Treatment with KaAPCs

Skin transplantation was performed as described by Garrod and Cahalan (31) with minor modifications. Briefly, dorsal tissue of ear was prepared from male C57BL/6J mice and then grafted onto the dorsal flank area of male bm1 mice under anesthesia. BAND AID^®^ styptic plaster was placed over the grafts, and an adhesive bandage was applied for 7 days. Each grafted mouse was then housed individually. After removing the styptic plaster, all of the recipients for which the operation was successful were randomly assigned to one of three groups and injected (*via* tail vein) with KaAPCs, anti-Fas-MPs or sterile 0.1 M PBS on days 9, 11, and 13 posttransplantation (1 × 10^7^ MPs/mouse/time point). The signs of allograft rejection were monitored daily. Photographs of alloskin graft were taken every 2 days. Grafts were defined as rejected when less than 10% of the graft remained viable.

### Histological and Immunohistochemical Analyses of Alloskin Graft

Full-thickness alloskin grafts were dissected from recipients on day 20 after transplantation and embedded in paraffin. Sections were prepared with the thickness of 10 µm and incubated with H-2K^b^-Ig dimer, rat anti-mouse CD4 (clone GK1.5, eBiosciences, San Diego, CA, USA), rat anti-mouse CD8 (clone H35-17.2, eBiosciences), or rat IgG2b/mouse IgG1 isotype control (eBiosciences) at 4°C overnight and further incubated with biotinylated mouse anti-rat IgG (eBiosciences) or biotinylated rat anti-mouse IgG1 (clone A85-1, BD Biosciences) for 1 h at RT. After washing, the sections were visualized using the ABC kit (Boster Biological Technology, Ltd., Wuhan, China). Hematoxylin and eosin (H&E) was also carried out routinely. Each section was scanned under the microscope (Nikon). The mean percentage of positive-staining cells was obtained by counting five separated fields (200×) using Image-Pro Plus software (Media Cybernetics, Rockville, MD, USA).

### Detection of Alloantigen-Specific CD8^+^ T Cells

Peripheral blood was collected from orbital veins of bm1 recipient mice on days 8 and 15 posttransplantation and processed into a lymphocyte suspension. Splenocytes were also collected on day 15 from bm1 recipient mice. Cells were then blocked with anti-mouse CD16/CD32 (clone 2.4G2, BD Biosciences) (1 µg/10^6^ cells) for 30 min followed by incubation with the mixture of peptide-unloaded H-2K^b^-Ig dimer and PE-anti-mouse IgG1 (clone A85-1, BD Biosciences) for 1 h at 4°C. After washing, FITC-anti-mouse CD8a (clone 53-6.7, eBiosciences) and allophycocyanin (APC)-labeled anti-mouse CD3e (clone 145-2C11, eBiosciences) were added for further 30-min incubation. Finally, cells were analyzed by flow cytometry as described.

### *In Vivo* Fluorescence Imaging

The KaAPCs, anti-Fas-MPs, and Blank-MPs that were encapsulated with ICG were injected *via* the tail vein into the grafted bm1 mice (1 × 10^7^ MPs/mouse), respectively, on day 9 after skin transplantation. The mice were then anesthetized by isoflurane inhalation and imaged using the Maestro *in vivo* fluorescence imaging system (Cri Inc., Woburn, MA, USA) at various time points. Images were captured at an excitation wavelength of 635 nm and at an emission wavelength of 665–695 nm with an exposure time of 40 s. At 2 h after injection, the heart, liver, kidneys, lungs, spleen, and lymph nodes (LNs) were surgically dissected for *ex vivo* imaging.

### Tissue Distribution of KaAPCs and Histological Analyses

The KaAPCs were injected *via* the tail vein into the grafted bm1 mice (1 × 10^7^ MPs per mouse) on day 9 after skin transplantation. 30 min later, peripheral blood was collected from orbital venous followed by Wright’s staining. At 1 h after injection, spleen and LNs were collected from the mice, processed into cell suspensions, and followed by Wright’s staining. In parallel, various organs and the full-thickness alloskin graft were dissected at 1-h time point and embedded in paraffin. Sections were then prepared with the thickness of 6 µm followed by H&E staining. Finally, the KaAPCs were visualized under optical microscope.

### Colocalization of KaAPCs with CD8^+^ T Cells *In Vivo*

Killer artificial antigen-presenting cells coupled with PE-streptavidin (PE-KaAPC) were injected (*via* tail vein) into recipient mice on days 9, 11, and 13 posttransplantation as described. Spleen and LNs were collected at 12 h after the final injection. Each spleen was cut in half. The LNs and half of spleen were processed into cell suspensions and acquired on a FACSCalibur flow cytometer (BD Biosciences).

Another half of spleen was embedded in freezing medium (O.C.T., Sakura Finetek Inc., Torrance, CA, USA), snap-frozen in liquid nitrogen and stored at −80°C until use. About 10 µm-thickness sections were prepared and incubated with FITC-anti-mouse CD8a (1:150 dilution) for 1 h at RT and stained with DAPI (Sigma-Aldrich) for 5 min followed by imaging under confocal laser scanning microscopy (Olympus).

### Mixed Lymphocyte Reaction (MLR) and T-Cell Proliferation Assay

Splenocytes were prepared from recipient bm1 mice on day 20 posttransplantation, labeled with carboxyfluorescein succinimidyl ester (CFSE, Sigma-Aldrich), and seeded into round-bottom 96-well plates (BD Falcon) as responder cells (1 × 10^5^ cells/well), then co-incubated with stimulator splenocytes, pretreated with Mitomycin C (Sigma-Aldrich), from donor C57BL/6J mice (1 × 10^5^ cells/well). Cells were cocultured in complete RPMI 1640 medium (Gibco BRL) at 37°C, 5% CO_2_, and humidified conditions for 7 days, then stained with APC-anti-mouse CD3e for 30 min at 4°C, and analyzed by Flow cytometry. A total of 2 × 10^5^ events were counted for each sample. Cell divisions were demarcated according to CFSE-staining brightness. A third-party MLR was also performed in a similar manner with the stimulator splenocytes from BALB/c mice (H-2K^d^) rather than C57BL/6J mice.

### Analysis of T Cells Apoptosis

Splenocytes were prepared from recipient mice on day 15 posttransplantation, incubated with APC-anti-mouse CD3e or APC-anti-mouse CD8a (clone 53-6.7, eBiosciences) for 30 min at 4°C and then stained with Annexin V and propidium iodide (PI) according to the manufacturer’s protocol (Dead Cell Apoptosis Kit, Invitrogen, Carlsbad, CA, USA) and analyzed by flow cytometry as described.

### Enumeration of Various Immune Cells in Spleen and Peripheral Blood

Splenocytes were harvested from recipient mice on day 15 posttransplantation and stained with APC-anti-mouse CD3e, FITC-anti-mouse CD8a, PE-labeled anti-mouse CD4 (clone GK1.5, eBiosciences), FITC-anti-mouse CD19 (clone MB19-1, Biolegend, San Diego, CA, USA), and FITC-anti-mouse NK1.1 (clone PK136, Biolegend), respectively, for 30 min at 4°C. After washing with PBS, cells were analyzed by flow cytometry as described. In parallel, peripheral blood was collected from orbital venous of recipient mice on days 11, 13, 15, and 45 after transplantation, blood cells were enumerated routinely by automated hematology analyzer (Sysmex XE-2100, Kobe, Japan).

For the detection of regulatory T cells (Tregs), spleen and LNs of recipient mice were collected on day 20 posttransplantation and processed into cell suspensions. Tregs were detected according to the protocol of Mouse Regulatory T Cell Staining Kit (eBiosciences) and analyzed by flow cytometry.

### Tumor Cells Challenge

On day 3 post-skin transplantation, the recipient bm1 mice were inoculated s.c. in the right groin with B16F10 melanoma cells at a dose of 1 × 10^6^ cells/mouse. Then, the mice were randomly assigned to one of three groups and administered (*via* tail vein) with KaAPCs, anti-Fas-MPs, or PBS on days 9, 11, and 13 after transplantation (1 × 10^7^ MPs/mouse/time point). Tumor size was measured daily using a caliper, and the products of perpendicular diameters were determined. Mice were sacrificed when tumor size reached to 250 mm^2^.

### Cytotoxicity Assay of NK Cells

Splenocytes were prepared from recipient mice on day 15 posttransplantation. A total of 1 × 10^7^ cells were labeled with CFSE as described and used as effector cells. Yac-1 cells were used as target cells. Effector cells were cocultured with target cells (2 × 10^5^ cells/well) at indicated ratios of effector to target in round-bottom 96-well plates in complete RPMI 1640 medium at 37°C, 5% CO_2_, and humidified conditions for 5 h, then harvested and analyzed by flow cytometry after staining with 7-amino-actinomycin D (7-AAD, eBiosciences). NK activity was calculated as the percentage of 7-AAD-positive cells within CFSE-negative cell population.

### Evaluation of Organ Toxicity

Peripheral blood was collected from orbital venous of recipient mice on days 15 and 45 after transplantation, the routine biochemical parameters evaluating liver function and kidney function were detected by automated biochemistry analyzer (Dimension Vista 1500, Siemens Healthcare Diagnostics Inc., Newark, DE, USA). In parallel, heart, liver, spleen, lung, and kidney were collected from recipient mice on days 15, 30, and 45 after transplantation, fixed in 4% formaldehyde and embedded in paraffin. Sections were prepared with the thickness of 6 µm followed by H&E staining routinely.

### Statistical Analyses

Statistical analyses were performed using the GraphPad Prism 6.0 (GraphPad, La Jolla, CA, USA). To determine the graft survival curve, a Kaplan–Meier graph was constructed, and a log-rank comparison of the groups was used to calculate the *p* values. The tumor sizes were analyzed using the Wilcoxon signed rank test. For other experiments, a two-tailed unpaired Student’s *t*-test was used to determine significant differences across groups. All data are presented as the mean ± SD. *p* Values < 0.05 were considered significant.

## Results

### Characterization of PLGA MPs and Phenotypic Analyses of KaAPCs

**Figure 1:**
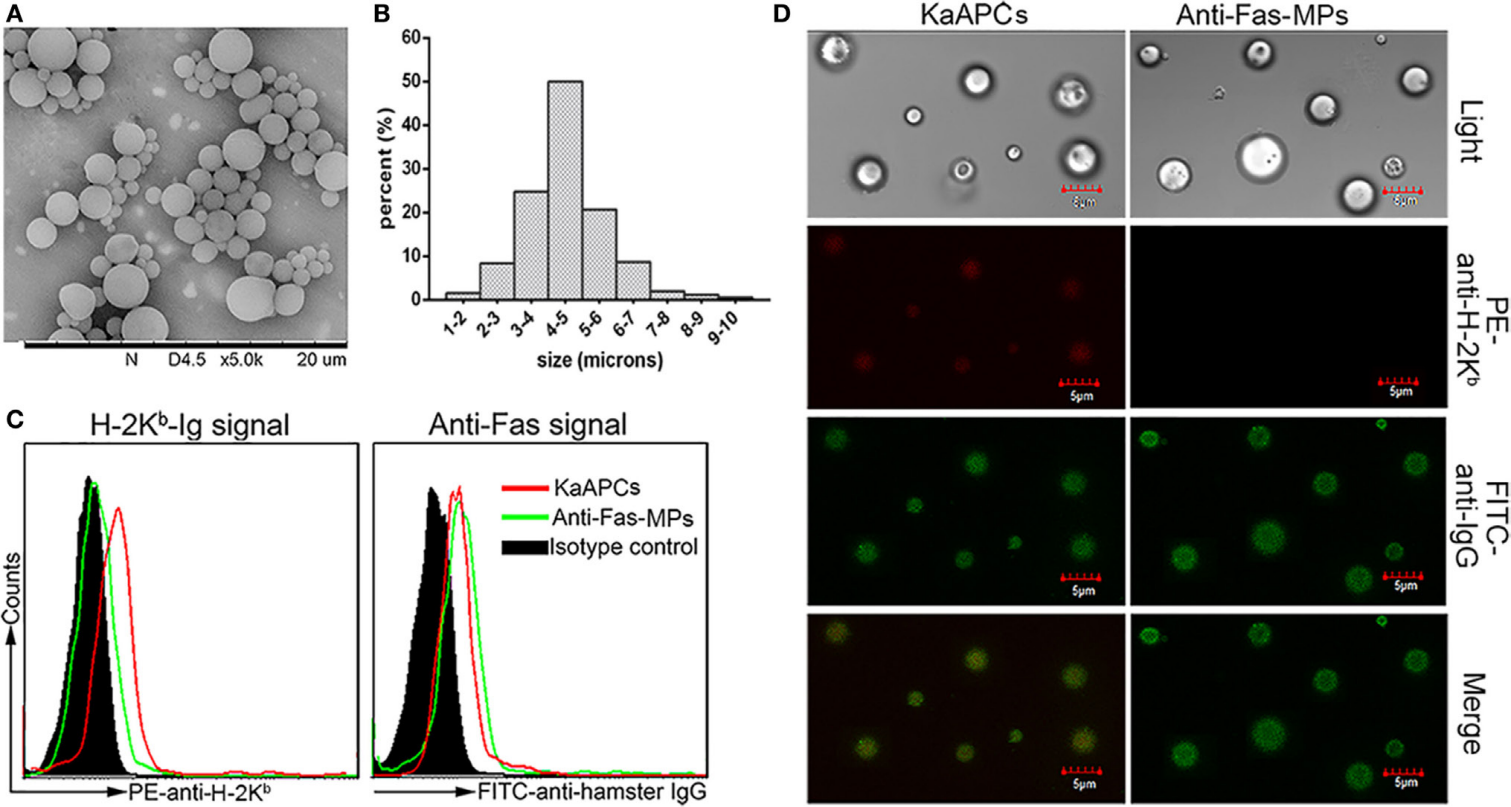
Generation and characterization of poly lactic-co-glycolic acid microparticles (PLGA MPs) and killer artificial antigen-presenting cells (KaAPCs). **(A)** Representative scanning electron microscopy image of PLGA MPs. **(B)** Size distribution of PLGA MPs. After staining with R-phycoerythrin (PE)-anti-H-2K^b^ and fluorescein-5-isothiocyanate (FITC)-anti-hamster IgG (binds to anti-Fas) monoclonal antibodies, KaAPCs and anti-Fas-MPs were analyzed by flow cytometry **(C)** and confocal laser scanning microscope **(D)**.

The PLGA MPs generated here displayed a spherical shape with a smooth surface morphology (Figure 1A). Size analysis showed a diameter range from 1 to 10 µm, and most of these MPs were 4–5 µm in diameter (Figure 1B). The mean zeta potential was 60.6 ± 5.9 mV as detected by the PALS zeta instrument (data not shown). As detected by flow cytometry, the KaAPCs displayed both H-2K^b^-Ig and anti-Fas signals immobilized onto the PEI-coated PLGA MPs, whereas the anti-Fas-MPs only showed anti-Fas signal (Figure 1C). Furthermore, confocal images also confirmed the correct phenotype of KaAPCs and anti-Fas-MPs (Figure 1D). Each batch of KaAPCs and control MPs was routinely evaluated in a same way to confirm the immobilization of two signals prior to use. According to the dot plots of KaAPCs stained by PE-anti-H-2K^b^ (or APC-anti-mouse IgG1) and FITC-anti-hamster IgG, nearly 70% KaAPCs showed double-positive staining (Figure S1 in Supplementary Material).

### KaAPCs Treatment Markedly Prolongs Alloskin Graft Survival and Reduces Local Allorejection Reaction

**Figure 2:**
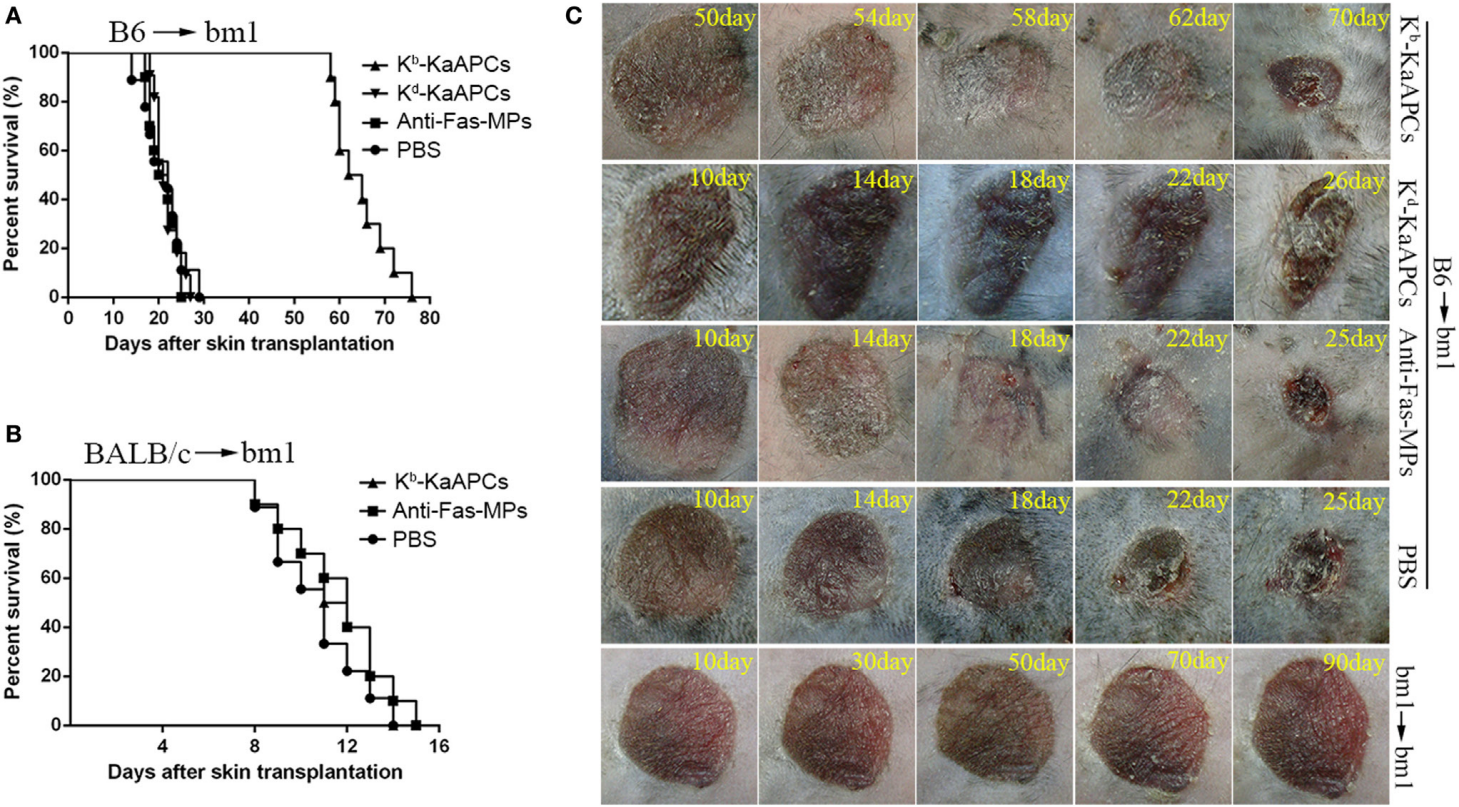
Killer artificial antigen-presenting cells (KaAPCs) prolong alloskin graft survival and reduce local allograft rejection. Ear dorsal tissues of C57BL/6J (B6) mice were grafted onto the dorsal of bm1 mice and followed by *i*.*v*. injection of K^b^-KaAPCs, K^d^-KaAPCs, anti-Fas-MPs (1 × 10^7^ MPs/mouse/time point), or PBS on days 9, 11, and 13 posttransplantation. **(A)** Kaplan–Meier survival plots for alloskin grafts in the bm1 mice grafted with ear skin of B6 mice in each treatment group. K^b^-KaAPCs mean the poly lactic-co-glycolic acid microparticles (PLGA MPs) co-displaying H-2K^b^-Ig dimers and anti-Fas monoclonal antibodies (mAbs); K^d^-KaAPCs mean the PLGA MPs co-displaying H-2K^d^-Ig dimers and anti-Fas mAbs; anti-Fas-MPs mean the PLGA MPs displaying only anti-Fas mAbs. **(B)** Kaplan–Meier survival plots for alloskin grafts in the bm1 mice grafted with ear skin of BALB/c mice, a third-party alloskin transplant model followed by treatment as described on days 5, 7, and 9 days posttransplantation. **(C)** Representative pictures of alloskin grafts on the indicated days in the KaAPC group and control groups. The bm1 autograft transplantation was performed to assure a correct transplant procedure.

C57BL/6J mice (H-2K^b^) and bm1 mice (H-2K^bm1^) were used as donor and recipient, respectively, to establish transplant model of alloskin graft and followed by intravenous injections of KaAPCs, anti-Fas-MPs, or PBS on days 9, 11, and 13 after transplantation. As shown in Figure 2A, the infusions of H-2K^b^ alloantigen-presenting KaAPCs (K^b^-KaAPCs) prolonged allograft survival for 43 days with a median survival time (MST) of 63.5 days. In contrast, the MST of anti-Fas-MPs and PBS group was 22 and 20.5 days, respectively. The differences between the KaAPC group and control groups presented *p* values less than 0.001 as determined by the log-rank test. More importantly, the H-2K^d^ alloantigen-presenting KaAPCs (K^d^-KaAPCs), which displaying a non-cognate alloantigen in this transplantation model, did not prolong alloskin survival, with an MST of 21 days (Figure 2A). Meanwhile, a third-party alloskin murine model was established by grafting the ear skin from BALB/c mice (H-2K^d^) onto bm1 mice in a same manner. The K^b^-KaAPCs administration did not lead to an obvious extension of alloskin survival as compared with the control groups in the third-party model (Figure 2B). Representative pictures for alloskin grafts of each group were shown in Figure 2C. There was no rejection occurring in the bm1 autograft transplantation group (Figure 2C, bottom row). The representative pictures of alloskin grafts in the third-party model were presented in Figure S2 in Supplementary Material.

**Figure 3:**
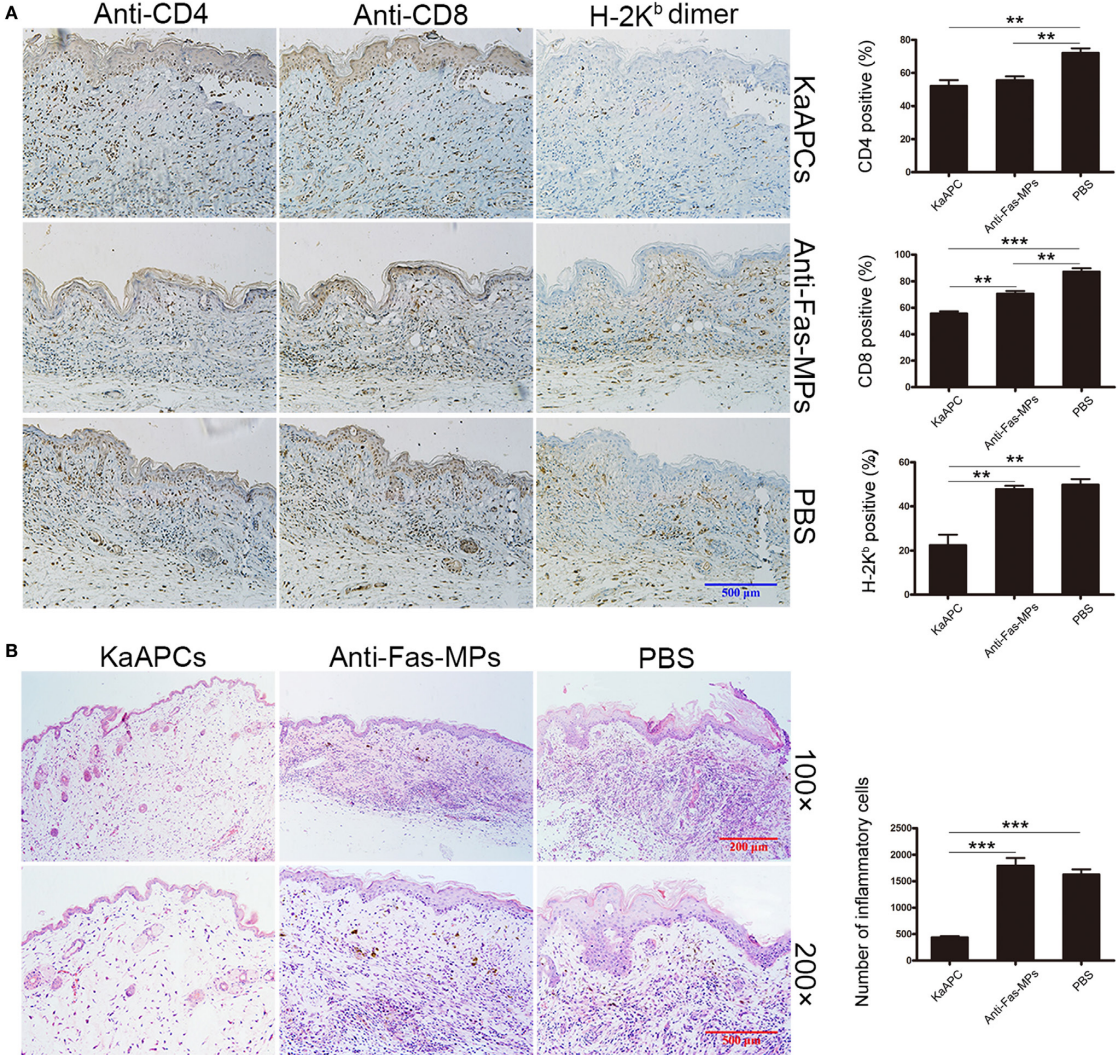
Killer artificial antigen-presenting cells (KaAPCs) reduce the local infiltration of alloreactive T cells and inflammatory cells in allograft. On day 20 posttransplantation (7 days after the final treatment), alloskin grafts were collected from recipients in each treatment group and embedded in paraffin. Allograft sections were prepared. **(A)** IHC analyses. Sections were incubated with H-2K^b^-Ig dimer, anti-mouse CD4, anti-mouse CD8, or IgG2b/isotype control monoclonal antibodies (mAbs) and then stained with biotinylated secondary antibodies followed by visualization using an ABC kit. **(B)** Hematoxylin and eosin staining. The IHC images were presented at 200× magnifications. Representative sections were selected from three to five individual mice. KaAPCs mean the K^b^-KaAPCs co-displaying H-2K^b^-Ig dimers and anti-Fas mAbs. **p < 0.01, ***p < 0.001.

Alloskin specimens were collected from each group on day 20 posttransplantation (7 days after the final injection of KaAPCs). As indicated by H-2K^b^-Ig dimer *in situ* staining, a remarkable reduction of H-2K^b^ alloantigen-reactive T cells was observed in the alloskin sections from the KaAPCs group (K^b^-KaAPCs), compared to the control groups (Figure 3A). The local infiltration of CD8^+^ T cells, but not CD4^+^ T cells, in allograft was relatively decreased after KaAPCs treatment (Figure 3A). Similarly, only weak inflammatory infiltration was found in the alloskin sections from the KaAPCs group while a strong local inflammation appeared in the control groups (Figure 3B).

### KaAPCs Selectively Deplete H-2K^b^ Antigen-Alloreactive CD8^+^ T Cells *In Vivo*

**Figure 4:**
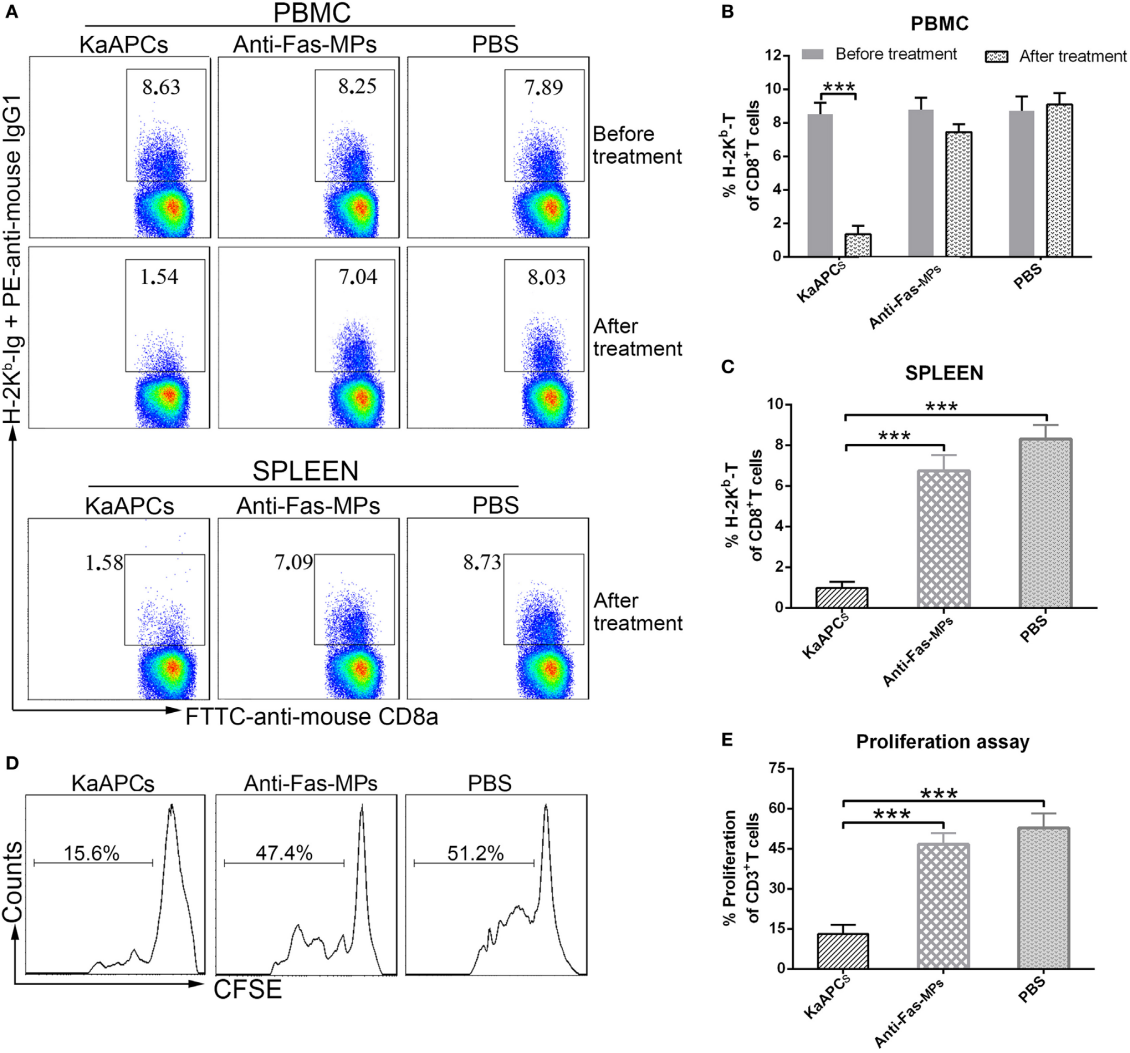
Killer artificial antigen-presenting cells (KaAPCs) deplete alloreactive T cells *in vivo* and inhibit the anti-donor alloreactivity of recipient T cells. Recipient bm1 mice were treated with KaAPCs, anti-Fas-MPs, or PBS on days 9, 11, and 13 after transplantation. Peripheral blood was collected on day 8 (1 day before treatment) and day 15 (2 days after treatment), and spleens were also collected on day 15. **(A–C)** H-2K^b^-Ig dimers staining and flow cytometry. The representative dot plots were presented in panel **(A)** and were gated on CD8^+^ T cell population. Infusions of KaAPCs resulted in a marked reduction of H-2K^b^-alloreactive CD8^+^ T cells in the CD8^+^ T cell populations from peripheral blood **(B)** and spleen **(C)**. **(D,E)** Anti-donor mixed lymphocyte reaction assays. Splenocytes from the recipient bm1 mice in each treatment group were labeled with carboxyfluorescein succinimidyl ester (CFSE) and cocultured with mitomycin C-treated splenocytes, which derived from donor C57BL/6J mice, in 96-well microplates for 7 days. The proliferation percentage of recipient CD3^+^ T cells was determined according to cell divisions **(D)**. KaAPCs treatment inhibited the anti-donor alloreactivity of recipient CD3^+^ T cells **(E)**. *n* = 4–6 mice in each group. ***p < 0.001.

The frequencies of H-2K^b^ antigen-alloreactive CD8^+^ T cells in the peripheral blood and spleen of recipients were detected by H-2K^b^-Ig dimer staining plus flow cytometry. Representative dot plots were presented in Figure 4A. The proportion of H-2K^b^-alloreactive CD8^+^ T cells in CD8^+^ T cell population of peripheral blood was 8.46 ± 0.61% on day 8 (before treatment) but markedly reduced to 1.45 ± 0.57% (a 83% decrease) on day 15 (after treatment) in the KaAPCs group. In contrast, the frequencies of H-2K^b^-alloreactive CD8^+^ T cells in the anti-Fas-MPs group or PBS group did not show significant changes before and after treatment (Figure 4B). Consistently, about 80% reduction of H-2K^b^-alloreactive CD8^+^ T cells was also found in the CD8^+^ T cell population from splenic cell suspension on day 15 in the KaAPCs group, as compared with the control groups (Figure 4C). Notably, the mean percentage of H-2K^b^-alloreactive CD8^+^ T in the CD8^+^ T cell population of peripheral blood mononuclear cells (PBMCs) showed no statistical increase on days 8 and 15 in the PBS group and was similar to that in the CD8^+^ T cell population of spleen cells (SPCs) in PBS group.

In addition, PBMCs of naïve bm1 mice before transplantation were also stained by the H-2K^b^-Ig dimer to detect the frequency of H-2K^b^-alloreactive T cells. As shown in Figure S3 in Supplementary Material, H-2K^b^-Ig dimer mainly stained with CD8^+^ T cells with a percentage of 0.75 ± 0.24% in CD3^+^ T cell population or 3.01 ± 0.73% in CD8^+^ T cell population, and very weak staining with CD4^+^ T cells (CD8^-^/CD3^+^) could be found (0.16 ± 0.07% in CD3^+^ T cell population). This frequency of H-2K^b^-alloreactive CD8^+^ T cells in naïve bm1 mice was nearly 2.8-fold lower than that after transplantation (8.46 ± 0.61% on day 8). As a negative control of H-2K^b^-Ig dimer staining, PBMCs of naïve C57BL/6 mice (H-2^b+^) were also stained by H-2K^b^-Ig dimer, but only background staining with CD8^+^ T cells was found.

Consistent with the nearly 80% reduction of H-2K^b^-alloreactive CD8^+^ T cells in the spleen of recipient mice, the *ex vivo* alloreactivity of recipient T cells was also markedly decreased in an MLR assay. Representative histograms of cell divisions were exhibited in Figure 4D. As shown in Figure 4E, the recipient CD3^+^ T cells from the KaAPCs group showed nearly 76% reduction of proliferation in response to donor splenocytes, relative to the recipient T cells from the control groups. Furthermore, the reduction of allo-proliferation was mainly contributed to the recipient CD8^+^ T cells, not CD4^+^ T cells (Figure S4 in Supplementary Material).

**Figure 5:**
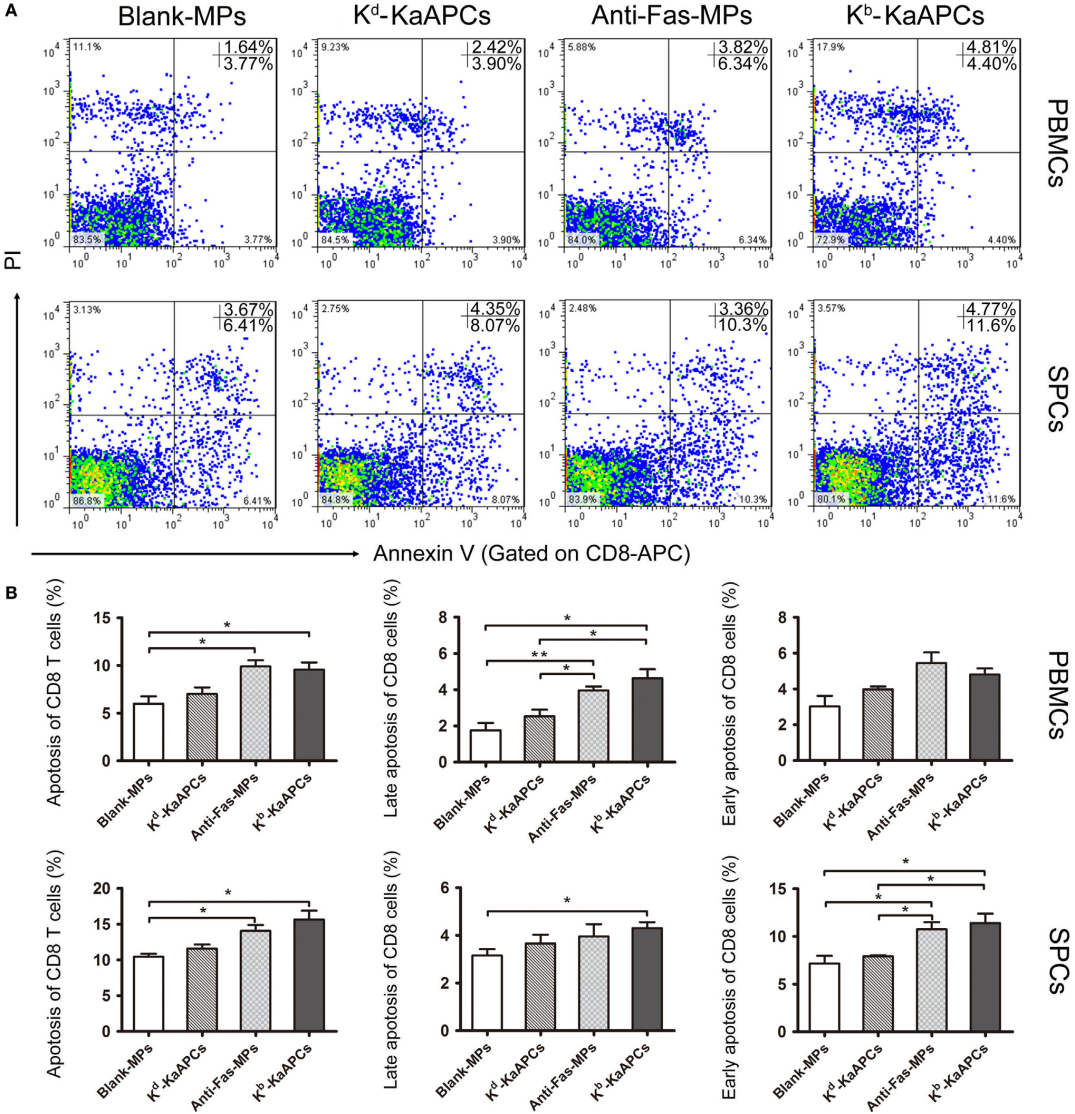
Killer artificial antigen-presenting cells (KaAPCs) induce apoptosis of CD8^+^ T cells *in vivo*. Grafted bm1 mice were injected *via* the tail vein with K^b^-KaAPCs, K^d^-KaAPCs, anti-Fas-MPs, or Blank-MPs on days 9, 11, and 13 after transplantation. Peripheral blood and spleen were collected on day 15 and processed into single cell suspensions followed by allophycocyanin-anti-mouse CD8a and Annexin V/propidium iodide (PI) staining for apoptosis analyses. **(A)** The representative dot plots for apoptosis of CD8^+^ T cells in peripheral blood mononuclear cells (PBMCs) and spleen cells (SPCs) of each group. **(B)** The frequencies of apoptotic CD8^+^ T cells in PBMCs and SPCs of each group. Data were displayed as the mean ± SD. *n* = 3–4 mice in each group. *p < 0.05, **p < 0.01.

To elucidate the mechanism by which KaAPCs reduced H-2K^b^-alloreactive CD8^+^ T cells, the apoptosis of CD8^+^ T cells in peripheral blood and spleen was analyzed on day 15 (2 days after the final injection of KaAPCs). As shown in Figure 5, the mean percentage of apoptotic CD8^+^ T cells increased from 5.8 ± 0.6 to 9.2 ± 0.4% in peripheral blood and from 10.5 ± 1.3 to 15.8 ± 1.8% in spleen (>50% elevation) as compared with the Blank-MPs group. In contrast, the non-cognate K^d^-KaAPCs only showed little higher apoptosis of CD8^+^ T cells than the Blank-MPs group without significant difference. As a non-targeting killer MPs control, anti-Fas-MPs also led to the increase of apoptotic CD8^+^ T cells with a frequency similar to the KaAPCs group (Figure 5B) but did not result in obvious decrease of H-2K^b^-alloreactive CD8^+^ T cells when compared with the PBS group (Figures 4A–C).

### *In Vivo* Tracking and Tissue Distribution of KaAPCs

**Figure 6:**
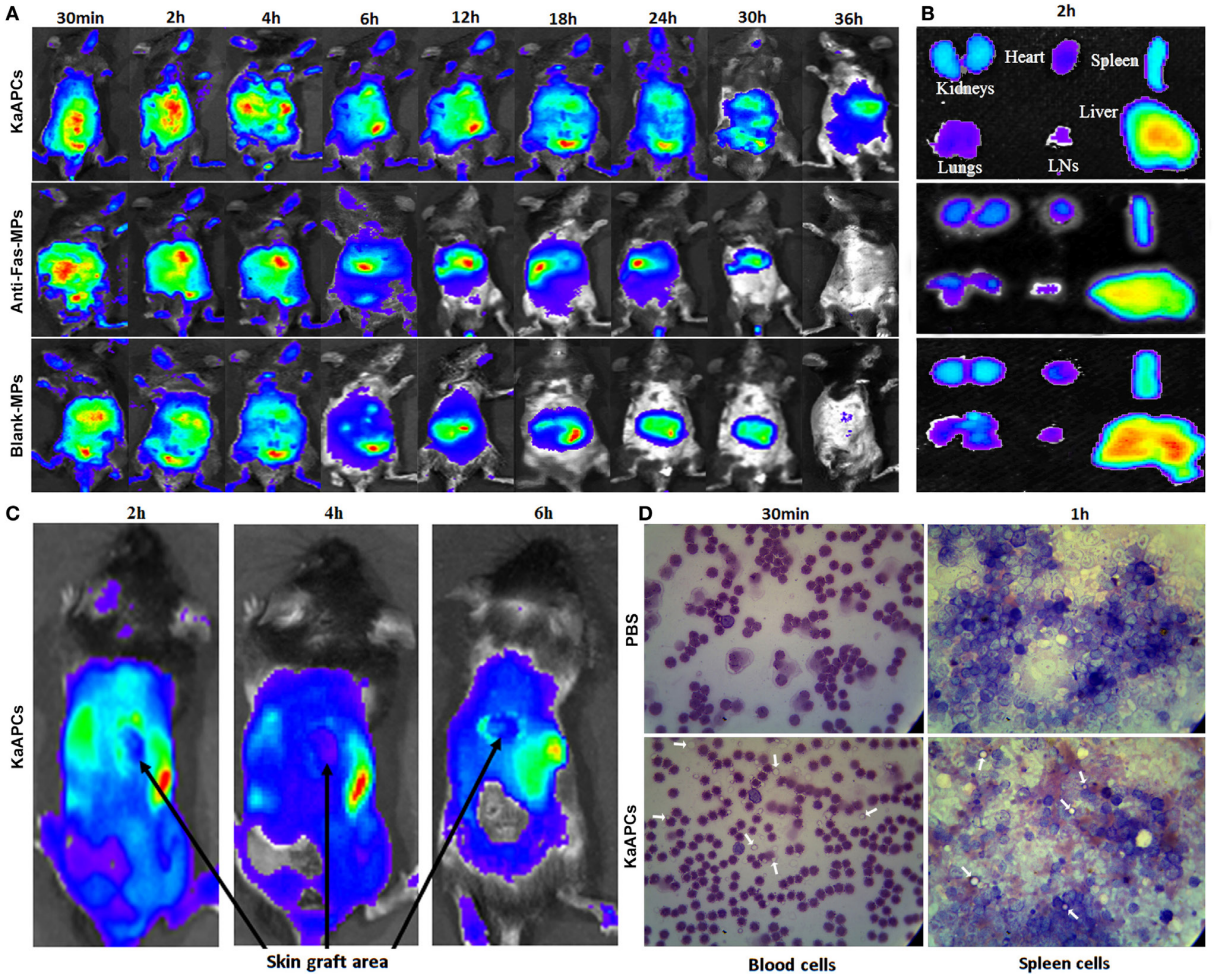
*In vivo* tracking and tissue distribution of killer artificial antigen-presenting cells (KaAPCs). Grafted bm1 mice were injected *via* the tail vein with indocyanine green-encapsulated KaAPCs, anti-Fas-MPs, or Blank-MPs. Fluorescence images were then acquired using the Maestro *in vivo* imaging system at different time points. **(A)** Whole-body fluorescence images for *in vivo* tracking of KaAPCs and the control microparticles (MPs). **(B)** Distribution of KaAPCs and control MPs in the excised organs 2 h after injection as displayed by *ex vivo* imaging. **(C)** Few KaAPCs circulated into the location of alloskin graft at 2-, 4-, and 6-h time points, as analyzed by whole-body fluorescence imaging. **(D)** Wright’s staining for peripheral blood cells and spleen cells (SPCs). KaAPCs were observed in the blood cells suspension at 30-min time point and in the SPCs’ suspension at 1-h time point after *i*.*v*. injection in the KaAPCs group. Meanwhile, no KaAPC was found in the PBS injection group. White arrows point at the KaAPCs.

To determine the fate of KaAPCs *in vivo* after *i*.*v*. administration, grafted bm1 mice were injected *via* the tail vein with ICG-encapsulated KaAPCs, anti-Fas-MPs, or Blank-MPs. Whole-body fluorescence imaging showed that fluorescent KaAPCs rapidly and selectively accumulated in the liver, spleen, kidney, lung, LNs, and heart. The fluorescent intensity in mice was the strongest 2–4 h after injection, with a retention time up to 36 h (Figure 6A, upper panel). As controls, both ICG-anti-Fas-MPs and ICG-Blank-MPs showed the *in vivo* trafficking similar to KaAPCs, but a shorter retention time (30 h) (Figure 6A, middle and down panels). At 2-h time point after injection, the KaAPCs mainly accumulated in liver, spleen, and kidney as displayed by the *ex vivo* imaging of excised organs (Figure 6B). Of note is that no or very weak fluorescent signal of KaAPCs was observed in the location of alloskin graft at 2-, 4-, and 6-h time points after KaAPCs *i*.*v*. injection (Figure 6C), suggesting that most of the cell-sized KaAPCs could not go into the alloskin graft through vascular circulation at least during the early 6 h.

Furthermore, the no-ICG KaAPCs or PBS was injected *i*.*v*. into the grafted bm1 mice on day 9 after skin transplantation. Wright’s staining showed that the KaAPCs were present in the cell suspensions of blood at 30-min time point and spleen at 1-h time point in the KaAPCs group (Figure 6D, lower panel), and absent in the cell suspensions of blood and spleen of the PBS group, a negative control (Figure 6D, upper panel). No KaAPCs were found in the LN cell population at 1-h time point after KaAPCs injection (data not shown). In parallel, histological analyses also displayed the presence of KaAPCs in the sections of liver, kidney, spleen, lung, and heart, but not alloskin graft at 1-h time point after *i*.*v*. injection (Figure S5 in Supplementary Material).

### KaAPCs Colocalize with CD8^+^ T Cells *In Vivo*

**Figure 7:**
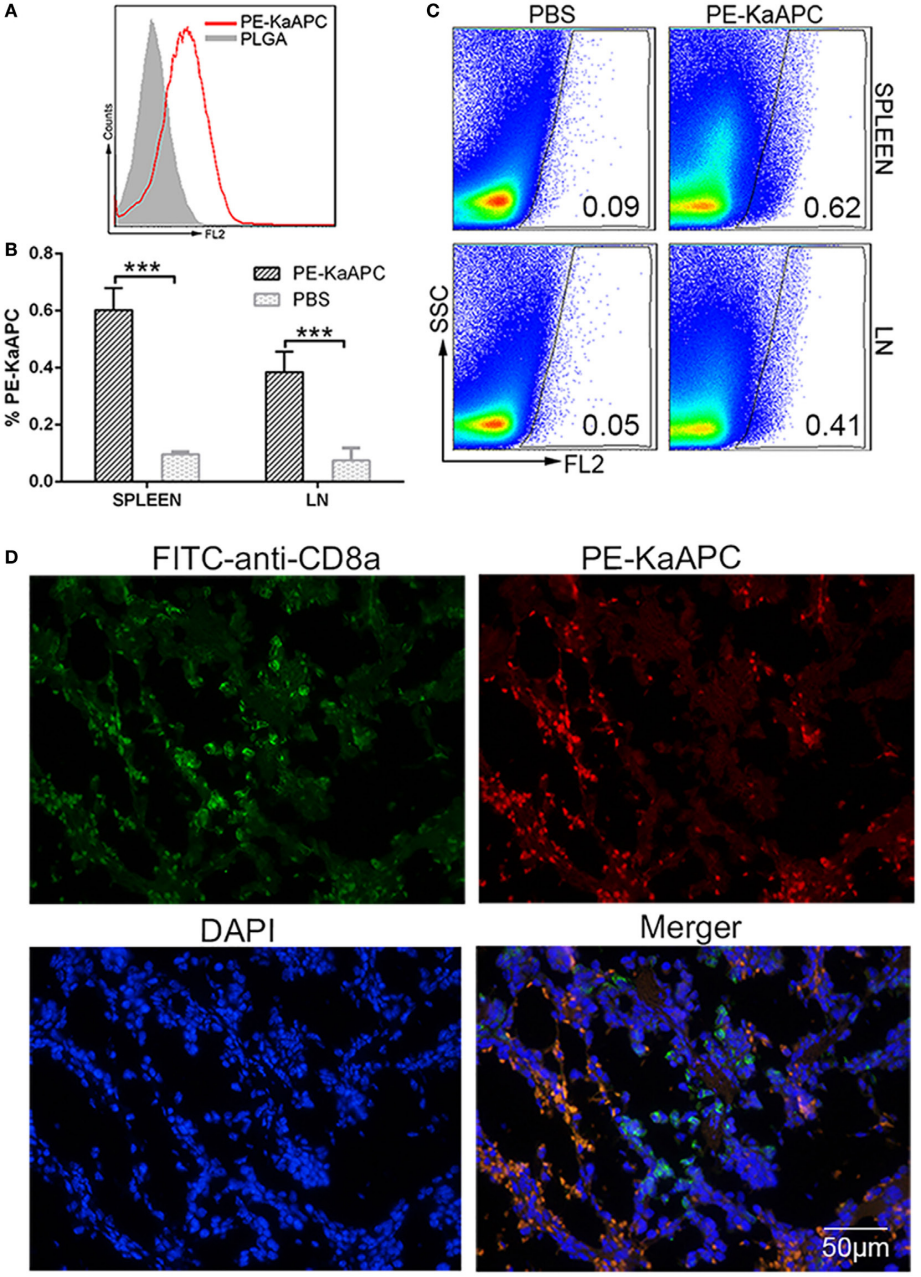
Killer artificial antigen-presenting cells (KaAPCs) circulate into secondary lymphoid organs and colocalize with CD8^+^ T cells. KaAPCs were coupled with R-phycoerythrin (PE)-labeled streptavidin and characterized by flow cytometry **(A)** and then injected *i*.*v*. into recipient bm1 mice on days 9, 11, and 13 after transplantation as described. At 12 h after the final injection, spleen and lymph nodes (LNs) were harvested from recipients. LNs and half of spleen were processed into single cell suspensions and freshly detected by flow cytometry without any staining. **(B)** A visible population of PE-KaAPCs was found in spleen and LNs, respectively. *n* = 3 mice in each group. Representative dot plots for flow cytometry analyses were presented in panel **(C)**. Another half of spleen was embedded into O.C.T. followed by frozen section preparation and IHC staining with fluorescein-5-isothiocyanate (FITC)-labeled-anti-mouse CD8a and DAPI. Confocal photomicrographs of PE-KaAPCs and CD8^+^ T cells in spleen section were presented in panel **(D)**, at 100× magnification. ***p < 0.001.

Phycoerythrin-coupled KaAPCs were prepared (Figure 7A) and injected into grafted bm1 mice on days 9, 11, and 13 after transplantation. A visible percentage of PE-KaAPCs was detected 12 h after the final injection, in the cell suspensions of spleen and LNs from recipients by flow cytometry (Figure 7B). Representative dot plots were showed in Figure 7C. The LN cells were a mixture of inguinal, axillary, and brachial LNs. Furthermore, PE-KaAPCs were found in the spleen tissue section and could colocalize with CD8^+^ T cells as revealed by confocal images (Figure 7D).

**Figure 8:**
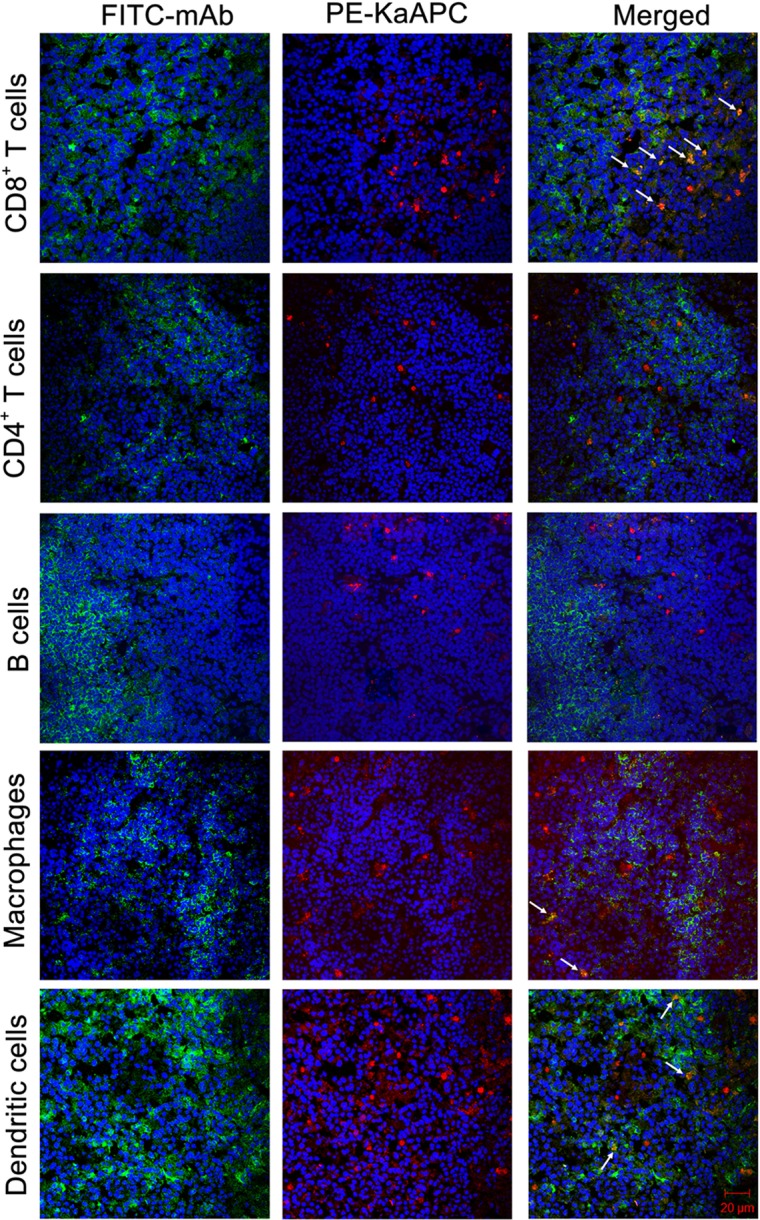
Confocol fluorescence imaging of killer artificial antigen-presenting cells (KaAPCs) with CD8^+^ T cells, CD4^+^ T cells, B cells, macrophages, and dendritic cells (DCs) in spleen section. R-Phycoerythrin (PE)-coupled KaAPCs were injected *i*.*v*. into the grafted bm1 mice on day 9, and spleens were collected 4 h later for the preparation of frozen sections. CD8^+^ T cells, CD4^+^ T cells, B cells, macrophage, and DCs were then stained by fluorescein-5-isothiocyanate (FITC)-labeled monoclonal antibodies (mAbs), respectively, and followed by confocol imaging in the marginal zone, red pulp, or white pulp of spleen section at 400× magnification. Many CD8^+^ T cells, macrophages, and DCs distributed in the marginal zones and red pulps, but most of B cells and CD4^+^ T cells could be observed in red pulps. Meanwhile, the PE-KaAPCs were mainly found in the marginal zones and red pulps.

To ascertain this colocalization, another independent experiment was carried out. PE-KaAPCs were injected *i*.*v*. into the grafted bm1 mice on day 9, and then spleens were collected 4 h later followed by the preparation of frozen sections and immunohistochemical staining. As shown in Figure 8, many CD8^+^ T cells, macrophages, and DCs could be observed in the marginal zone and red pulp of spleen while most of CD4^+^ T cells and B cells were absent in marginal zone. PE-KaAPCs mainly distributed in the red pulp and marginal zone and presented many co-localizations with CD8^+^ T cells, but much fewer co-localizations with CD4^+^ T cells, B cells, macrophage, and DCs. Also, no visible internalization or engulfment by macrophage and DCs was displayed in the spleen sections. These results may suggest the direct contacts of KaAPCs with CD8^+^ T cells in secondary lymphoid organ.

### KaAPCs Treatment Does Not Produce Obvious Bystander Killing to Immune Cells

**Figure 9:**
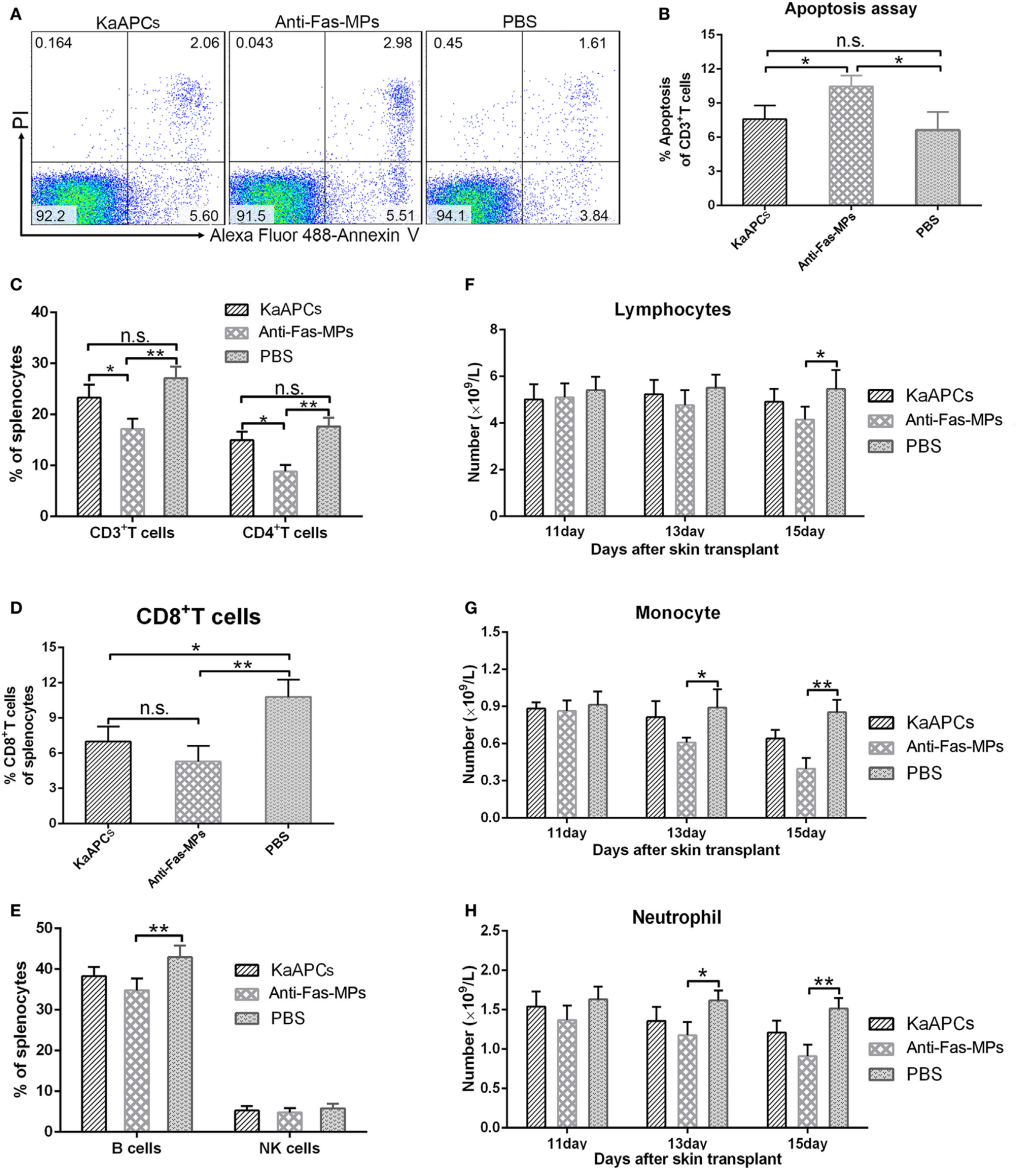
Killer artificial antigen-presenting cells (KaAPCs) do not produce obvious bystander killing to immune cells. After treatment with KaAPCs, anti-Fas-MPs, or PBS on days 9, 11, and 13 after transplantation, spleens were harvested on day 15, and peripheral blood was collected on days 11, 13, and 15 from recipients. Apoptosis of T cells was analyzed by Annexin V/PI staining. A variety of immune cells were enumerated by flow cytometry or automated hematology analyzer. The representative dot plots for apoptosis of T cells from spleens in each treatment group were presented **(A)**. KaAPCs treatment did not lead to obviously higher percentage of apoptosis in the T cell populations than the PBS treatment, but the percentage of apoptotic T cells in anti-Fas-MPs group was statistically higher than the KaAPCs group and PBS group **(B)**. In spleen cell suspensions, the percentages of CD3^+^ and CD4^+^ T cell populations **(C)**, CD8^+^ T cell populations **(D)**, and B cell and NK cell populations **(E)** in each treatment group were presented. In peripheral blood, injections of KaAPCs did not obviously decrease the amounts of lymphocytes **(F)**, monocytes **(G)**, and neutrophils **(H)** 2 days after each injection. *n* = 4–6 mice in each group at each time point. *p < 0.05, **p < 0.01.

On day 15 (2 days after the final injection of KaAPCs), infusion of KaAPCs did not lead to the obviously increased apoptosis in the T cell repertoires, but the percentage of apoptotic T cells in anti-Fas-MPs group was statistically higher than the KaAPCs group and PBS group (Figures 9A,B). Meanwhile, KaAPCs treatment did not result in the significant reduction of CD3^+^ and CD4^+^ T cells (Figure 9C), B cells and NK cells (Figure 9E) but elicited an obvious decrease of CD8^+^ T cells (Figure 9D) in the SPC suspensions. As a non-targeting control, the three infusions of anti-Fas-MPs caused a remarkable decrease of CD3^+^ T cells, CD4^+^ T cells, and B cells (Figures 9C–E). Representative flow cytometric diagrams of B cell and NK cell staining were presented in Figure S6 in Supplementary Material. Accordantly, on day 9, 13, and 15 (2 days after each injection), the numbers of lymphocytes, monocytes, and neutrophils in peripheral blood did not remarkably decreased in the KaAPCs group as detected by automated hematology analyzer but reduced in the anti-Fas-MPs group at some time points (Figures 9F–H). Furthermore, to get the results at longer time point, blood routine tests were performed on days 15 and 45 (2 and 32 days after the final injection) in another independent experiment. No obvious bystander killing to eight populations of non-pathogenic immune cells was found *in vivo* after KaAPCs treatment (Figure S7 in Supplementary Material). Notably, on day 20 (7 days after the final injection), a statistically higher proportion of CD4^+^/CD25^+^/Foxp3^+^ Tregs was found in LNs, but not in spleen, in the KaAPCs group as compared to the two control groups (Figure S8 in Supplementary Material).

### KaAPCs Treatment Does Not Cause Impairment of Overall Host Immune Function and Observable Organ Toxicity

The antitumor effect and reactivity against alloantigen could be used as surrogate markers for retention of overall host immune function (32). Here, the recipient T cells from the KaAPCs group and PBS group presented comparable proliferation levels in response to the SPCs from naïve BALB/c mice (Figures S9A,B in Supplementary Material), suggesting that the native T cell repertoires of recipient mice retained the third-party reactivity after KaAPCs treatment. In parallel, the similar cytolysis levels of recipient NK cells against Yac-1 lymphoma cells (Figure S9C in Supplementary Material) and comparable tumor growth in the B16 melanoma challenge (Figure S9D in Supplementary Material) were found in the KaAPCs group and PBS group.

The functions of liver and kidney of recipient mice were monitored at 2 and 32 days after the final injection of KaAPCs by routine biochemical tests. No significant impairment was found as compared with the two control groups (Figure S10 in Supplementary Material). Meanwhile, the histological H&E staining confirmed that KaAPCs treatment did not lead to visible pathological injuries in liver (Figure S11 in Supplementary Material), spleen (Figure S12 in Supplementary Material), kidney, heart, and lung (Figure S13 in Supplementary Material) on days 2, 17, and 32 after the final injection.

## Discussion

In this study, PEI-coated PLGA MPs were employed as cell-sized scaffold to co-present alloantigen and apoptosis-inducing molecule to alloreactive T cells for the treatment of alloskin rejection. After *i*.*v*. injection, the KaAPCs could circulate throughout vasculature into spleen, LNs, liver, kidney, lung, and heart, but most of KaAPCs did not go into the local allograft during the early 6 h; they could colocalize with CD8^+^ T cells along with few contacts with other immune cells and minor internalization by phagocytes in secondary lymphoid organ. The KaAPCs markedly depleted H-2K^b^ alloantigen-specific CD8^+^ T cells in bloodstream and spleen thereby leading to the decreased local infiltration of H-2K^b^ alloantigen-specific CD8^+^ T cells and inflammatory cells in alloskin graft, thus consequently prolonged allograft survival.

To define the possible mechanism by which KaAPCs deplete target T cells, apoptosis of CD8^+^ T cells was detected. In our previous works, OVA_257–264_-specific CD8^+^ T cells were used as target cells since their high frequency in transgenic OT-1 mice. Two infusions of the KaAPCs co-coupling H-2K^b^/OVA_257–264_ dimer and anti-Fas onto PLGA MPs led to 70% increase of apoptotic CD8^+^ T cells and around 60% reduction of OVA_257–264_-specific CD8^+^ T cells in OT-1 mouse. Conversely, the non-cognate PLGA-MPs co-presenting H-2K^b^/TRP2_257–264_ dimer and anti-Fas, OVA-MPs presenting only H-2K^b^/OVA_257–264_ dimer, and anti-Fas-MPs presenting only anti-Fas did not result in obviously increased apoptotic CD8^+^ T cells and reduced OVA_257–264_-specific CD8^+^ T cells (30). These data clearly document that the KaAPCs can physically deplete antigen-specific T cells mainly in an anti-Fas-dependent manner and an antigen-specific fashion either *in vivo* or *in vitro*. In the present study, the low-frequency H-2K^b^-alloreactive CD8^+^ T cells in the grafted bm1 mice were also markedly reduced (>80%) along with more than 50% increase of apoptotic CD8^+^ T cells after three infusions of H-2K^b^-targeted KaAPCs. In contrast, the non-cognate K^d^-KaAPCs only showed little higher apoptosis of CD8^+^ T cells than the Blank-MPs group. In addition, the K^d^-KaAPCs, anti-Fas-MPs, and a third-party alloskin transplantation model did not lead to a prolonged allograft survival. Thus far, both our previous work and the present data suggest the apoptosis-inducing and antigen-specific killing of KaAPCs to target cells. But we cannot eliminate the possibility of other immunoregulatory mechanisms occurring *in vivo*, such as the activation-induced cell death stimulated by target antigens onto KaAPCs, induction of Tregs, and inhibition of antigen-presenting cells.

Of note is that since the rapid development of biodegradable and biocompatible biomaterials, increasing attentions focus on the biomimetic MPs and nanoparticles as modulators in autoimmunity and transplantation by coating or encapsulating antigens and/or toxin (33–40). Presumably, these nanoscale particles will be mostly internalized by phagocytes or other cell types *in vivo* (41), then induce the tolerogenic APCs and Tregs, where after indirectly induce T cells tolerance (34, 37, 40–42). Relatively, cell-sized MPs (4–6 µm) present a reduced risk of engulfment by phagocytes (43, 44). Unlike these previous works, here the cell-sized KaAPCs co-displayed the intact MHC class I alloantigen molecules and apoptosis-inducing molecules, thus not only can directly present the alloantigen to T cell receptors onto alloreactive CD8^+^ T cells but also concurrently induce apoptosis, without the requirement of antigen processing by recipient antigen-presenting cells. Therefore, the KaAPCs mainly depleted the T cells participated in the direct pathway of allorecognition. Inevitably, indirect pathway may also involve in the KaAPCs immunotherapy at some extent since the possible phagocytosis occurring *in vivo*, especially at the later stage during the retention time of 36 h.

It is also important to note that a weak immunogenic murine model of alloskin transplantation was employed in this study. The H-2K^bm1^ is a variant allele, which differs from H-2K^b^ by seven nucleotides resulting in amino acid substitutions in three codons. Therefore, the bm1 and C57BL/6J mice are congenic strains mismatched only at K of b. But the single mutation between C57BL/6J and bm1 mice is sufficient to cause the acute rejections of donor skin graft with an MST of 19.5 ± 3 days (45, 46). The current study also showed an MST of 20.5 days. Thus, this single MHC-mismatched model is expected to maximally reveal the therapeutic effects of KaAPCs for alloskin rejection without the interference from the alloantigen responses against other mismatched MHC and minor histocompatibility loci, which will be helpful to the mechanism investigation. The alloskin graft from C57BL/6J mice was prolonged for 43 days in the bm1 mice in the present study, but only for 4–6 days in the BALB/c mice (26, 47), which are fully MHC-mismatched with C57BL/6J mice. The present data strongly documented the therapeutic potential of KaAPCs for allograft rejection. Nevertheless, to bring this goal to fruition, multiple MHC class I and II alloantigens mismatched from donor should be co-coupled on the KaAPCs in the translational studies from bench to bedside.

Finally, several technical notes are worthy of mentioning. In this weak immunogenic model, alloskin graft rejections appeared around day 14 after transplantation, thus the KaAPCs were administered three times with a 48-h interval before day 14 to prophylactically enact an inhibitory immune response by depleting the activated alloreactive T cells before they reach to the location of donor skin and cause acute immune rejections. Of note is that the administration before transplantation or on days 5, 7, and 9 or on days 7, 9, and 11 did not lead to the clinical efficacy (alloskin grafts survival and MST) as good as the present regimen in this single MHC-mismatched murine model. In addition, intravenous but not subcutaneous administration of the KaAPCs protected the alloskin rejection and prolonged the allograft survival in the present study and previous works. The injection *via* tail veil may facilitate the cell-sized KaAPCs circulating into spleen and LNs and helpful to interplay between KaAPCs and target T cells. The amount and ratios of anti-Fas and pMHC molecules coupled onto MPs and the dosages of KaAPCs have been titrated in our researches to achieve maximal antigen-specific apoptosis of target T cells with minimal bystander killing. In contrast, as a non-targeting control, the anti-Fas-MPs treatment led to the decrease of variable immune cells and the impairment of overall immune function of host. This non-specific killing of anti-Fas-MPs led to the reduction of CD8^+^ T cells and increase of apoptotic CD8^+^ T cells with a level similar to the KaAPCs group but did not result in obvious decrease of H-2K^b^-alloreactive CD8^+^ T cells. Therefore, the alloskin graft survival was not prolonged in the H-2K^b^-mismatched model by the non-targeting anti-Fas-MPs. In addition, the Blank-MPs which only blocked with BSA were also used to treat the grafted bm1 mice in some experiments and showed comparable effects with the PBS group (data no shown).

In conclusion, the capability of PLGA MP-based KaAPCs to selectively deplete alloreactive T cells for prolonging allograft survival was strongly documented. The *in vivo* mechanism of alloinhibition, tissue distribution, toxicity, and clearance were also initially characterized. These new evidences, although preclinical, demonstrate the high potential of the KaAPCs as a novel antigen-specific immunotherapy for the treatment of allograft rejection and autoimmunity.

## Ethics Statement

Animal welfare and experimental procedures were performed in accordance with the Guide for the Care and Use of Laboratory Animals (Ministry of Science and Technology of China, 2006) and were approved by the Animal Ethics Committee of Southeast University.

## Author Contributions

CS designed the research. WW and KS performed the main experiments of this study. ML performed the experiments evaluating the overall immune function of host. AZ was responsible for tissue section preparation, H&E staining, and pathological observation. LZ generated all of PLGA MPs and performed characterization. TX and XW were responsible for cell cultures, flow cytometry, and data analysis. CS and WW wrote the manuscript with discussions from all authors. WW and KS contributed equally to this work.

## Conflict of Interest Statement

The authors declare that the research was conducted in the absence of any commercial or financial relationships that could be construed as a potential conflict of interest.

## Abbreviations

KAPCs, killer antigen-presenting cells; KaAPCs, killer artificial antigen-presenting cells; pMHC, peptide–major histocompatibility complex; PLGA, poly lactic-co-glycolic acid; PEI, polyethylenimine; NHS, *N*-Hydroxysuccinimide; EDC, 1-ethyl-3-(3-dimethylaminopropyl) carbodiimide hydrochloride; ICG, indocyanine green; MLR, mixed lymphocyte reaction; CFSE, carboxyfluorescein succinimidyl ester; PE, R-phycoerythrin; FITC, fluorescein-5-isothiocyanate; APC, allophycocyanin; 7-AAD, 7-amino-actinomycin D; SEM, scanning electron microscopy; MP, microparticle; NP, nanoparticle.

## References

[1] Li CJ Li L Tacrolimus in preventing transplant rejection in Chinese patients – optimizing use. Drug Des Devel Ther (2015) 9:473–85. 10.2147/dddt.s41349 PMC4298305 25609922

[2] Masson P Henderson L Chapman JR Craig JC Webster AC Belatacept for kidney transplant recipients. Cochrane Database Syst Rev (2014) 11:Cd010699 10.1002/14651858.CD010699.pub2 PMC6486198 25416857

[3] Provenzani A Santeusanio A Mathis E Notarbartolo M Labbozzetta M Poma P Pharmacogenetic considerations for optimizing tacrolimus dosing in liver and kidney transplant patients. World J Gastroenterol (2013) 19(48):9156–73. 10.3748/wjg.v19.i48.9156 24409044 PMC3882390

[4] Kosiewicz MM Krishnan A Worthington MT Matriano JA Ross WG. B cells engineered to express Fas ligand suppress pre-sensitized antigen-specific T cell responses in vivo. Eur J Immunol (2002) 32(6):1679–87. 10.1002/1521-4141(200206)32:6<1679::AID-IMMU1679>3.0.CO;2-5 12115651

[5] Schutz C Hoves S Halbritter D Zhang HG Mountz JD Fleck M. Alloantigen specific deletion of primary human T cells by Fas ligand (CD95L)-transduced monocyte-derived killer-dendritic cells. Immunology (2011) 133(1):115–22. 10.1111/j.1365-2567.2011.03417.x 21342185 PMC3088973

[6] Hoves S Krause SW Schutz C Halbritter D Scholmerich J Herfarth H Monocyte-derived human macrophages mediate anergy in allogeneic T cells and induce regulatory T cells. J Immunol (2006) 177(4):2691–8. 10.4049/jimmunol.177.4.2691 16888031

[7] Matsue H Matsue K Walters M Okumura K Yagita H Takashima A. Induction of antigen-specific immunosuppression by CD95L cDNA-transfected ‘killer’ dendritic cells. Nat Med (1999) 5(8):930–7. 10.1038/11375 10426318

[8] Matsue H Matsue K Kusuhara M Kumamoto T Okumura K Yagita H Immunosuppressive properties of CD95L-transduced “killer” hybrids created by fusing donor- and recipient-derived dendritic cells. Blood (2001) 98(12):3465–72. 10.1182/blood.V98.12.3465 11719389

[9] Yolcu ES Askenasy N Singh NP Cherradi SE Shirwan H. Cell membrane modification for rapid display of proteins as a novel means of immunomodulation: FasL-decorated cells prevent islet graft rejection. Immunity (2002) 17(6):795–808. 10.1016/S1074-7613(02)00482-X 12479825

[10] Whartenby KA Straley EE Kim H Racke F Tanavde V Gorski KS Transduction of donor hematopoietic stem-progenitor cells with Fas ligand enhanced short-term engraftment in a murine model of allogeneic bone marrow transplantation. Blood (2002) 100(9):3147–54. 10.1182/blood-2002-01-0118 12384412

[11] Kusuhara M Matsue K Edelbaum D Loftus J Takashima A Matsue H. Killing of naive T cells by CD95L-transfected dendritic cells (DC): in vivo study using killer DC-DC hybrids and CD4(+) T cells from DO11.10 mice. Eur J Immunol (2002) 32(4):1035–43. 10.1002/1521-4141(200204)32:4<1035::AID-IMMU1035>3.0.CO;2-7 11920570

[12] Hoves S Krause SW Halbritter D Zhang HG Mountz JD Scholmerich J Mature but not immature Fas ligand (CD95L)-transduced human monocyte-derived dendritic cells are protected from Fas-mediated apoptosis and can be used as killer APC. J Immunol (2003) 170(11):5406–13. 10.4049/jimmunol.170.11.5406 12759415

[13] Buonocore S Flamand V Claessen N Heeringa P Goldman M Florquin S. Dendritic cells overexpressing Fas-ligand induce pulmonary vasculitis in mice. Clin Exp Immunol (2004) 137(1):74–80. 10.1111/j.1365-2249.2004.02514.x 15196246 PMC1809076

[14] Strauss G Osen W Knape I Jacobsen EM Muller SM Debatin KM. Membrane-bound CD95 ligand expressed on human antigen-presenting cells prevents alloantigen-specific T cell response without impairment of viral and third-party T cell immunity. Cell Death Differ (2007) 14(3):480–8. 10.1038/sj.cdd.4402019 16902496

[15] Sun W Adams RN Miagkov A Lu Y Juon HS Drachman DB. Specific immunotherapy of experimental myasthenia gravis in vitro and in vivo: the guided missile strategy. J Neuroimmunol (2012) 251(1–2):25–32. 10.1016/j.jneuroim.2012.06.007 22769060

[16] Chuang YH Suen JL Chiang BL Fas-ligand-expressing adenovirus-transfected dendritic cells decrease allergen-specific T cells and airway inflammation in a murine model of asthma. J Mol Med (2006) 84(7):595–603. 10.1007/s00109-006-0047-3 16565865

[17] Min WP Gorczynski R Huang XY Kushida M Kim P Obataki M Dendritic cells genetically engineered to express Fas ligand induce donor-specific hyporesponsiveness and prolong allograft survival. J Immunol (2000) 164(1):161–7. 10.4049/jimmunol.164.1.161 10605007

[18] Schutz C Oelke M Schneck JP Mackensen A Fleck M. Killer artificial antigen-presenting cells: the synthetic embodiment of a ‘guided missile’. Immunotherapy (2010) 2(4):539–50. 10.2217/imt.10.26 20636007 PMC2941805

[19] Greenwald RJ Freeman GJ Sharpe AH The B7 family revisited. Annu Rev Immunol (2005) 23:515–48. 10.1146/annurev.immunol.23.021704.115611 15771580

[20] Buonocore S Paulart F Le Moine A Braun M Salmon I Van Meirvenne S Dendritic cells overexpressing CD95 (Fas) ligand elicit vigorous allospecific T-cell responses in vivo. Blood (2003) 101(4):1469–76. 10.1182/blood-2002-07-2042 12393481

[21] Hermans IF Ritchie DS Yang J Roberts JM Ronchese F. CD8+ T cell-dependent elimination of dendritic cells in vivo limits the induction of antitumor immunity. J Immunol (2000) 164(6):3095–101. 10.4049/jimmunol.164.6.3095 10706699

[22] Yuan RR Wong P McDevitt MR Doubrovina E Leiner I Bornmann W Targeted deletion of T-cell clones using alpha-emitting suicide MHC tetramers. Blood (2004) 104(8):2397–402. 10.1182/blood-2004-01-0324 15217835

[23] Maile R Wang B Schooler W Meyer A Collins EJ Frelinger JA. Antigen-specific modulation of an immune response by in vivo administration of soluble MHC class I tetramers. J Immunol (2001) 167(7):3708–14. 10.4049/jimmunol.167.7.3708 11564786

[24] Hess PR Barnes C Woolard MD Johnson MD Cullen JM Collins EJ Selective deletion of antigen-specific CD8+ T cells by MHC class I tetramers coupled to the type I ribosome-inactivating protein saporin. Blood (2007) 109(8):3300–7. 10.1182/blood-2006-06-028001 17179221 PMC1852243

[25] Schutz C Fleck M Mackensen A Zoso A Halbritter D Schneck JP Killer artificial antigen-presenting cells: a novel strategy to delete specific T cells. Blood (2008) 111(7):3546–52. 10.1182/blood-2007-09-113522 18096763 PMC2275020

[26] Shen C He Y Cheng K Zhang D Miao S Zhang A Killer artificial antigen-presenting cells deplete alloantigen-specific T cells in a murine model of alloskin transplantation. Immunol Lett (2011) 138(2):144–55. 10.1016/j.imlet.2011.04.002 21513739

[27] Sahdev P Ochyl LJ Moon JJ. Biomaterials for nanoparticle vaccine delivery systems. Pharm Res (2014) 31(10):2563–82. 10.1007/s11095-014-1419-y 24848341 PMC4198431

[28] Ortega-Oller I Padial-Molina M Galindo-Moreno P O’Valle F Jodar-Reyes AB Peula-Garcia JM. Bone regeneration from PLGA micro-nanoparticles. Biomed Res Int (2015) 2015:415289. 10.1155/2015/415289 26509156 PMC4609778

[29] Ren H Han M Zhou J Zheng ZF Lu P Wang JJ Repair of spinal cord injury by inhibition of astrocyte growth and inflammatory factor synthesis through local delivery of flavopiridol in PLGA nanoparticles. Biomaterials (2014) 35(24):6585–94. 10.1016/j.biomaterials.2014.04.042 24811262

[30] Wang W Fang K Li MC Chang D Shahzad KA Xu T A biodegradable killer microparticle to selectively deplete antigen-specific T cells in vitro and in vivo. Oncotarget (2016) 7(11):12176–90. 10.18632/oncotarget.7519 26910923 PMC4914277

[31] Garrod KR Cahalan MD Murine skin transplantation. J Vis Exp (2008) 11:634 10.3791/634 PMC2582837 19066559

[32] Brandhorst G Weigand S Eberle C Raddatz D Karaus M Oellerich M CD4+ immune response as a potential biomarker of patient reported inflammatory bowel disease (IBD) activity. Clin Chim Acta (2013) 421:31–3. 10.1016/j.cca.2013.02.016 23485644

[33] Hotaling NA Tang L Irvine DJ Babensee JE Biomaterial strategies for immunomodulation. Annu Rev Biomed Eng (2015) 17:317–49. 10.1146/annurev-bioeng-071813-104814 26421896 PMC4798784

[34] Fisher JD Acharya AP Little SR. Micro and nanoparticle drug delivery systems for preventing allotransplant rejection. Clin Immunol (2015) 160(1):24–35. 10.1016/j.clim.2015.04.013 25937032 PMC4554802

[35] Hubbell JA Thomas SN Swartz MA Materials engineering for immunomodulation. Nature (2009) 462(7272):449–60. 10.1038/nature08604 19940915

[36] Getts DR Martin AJ McCarthy DP Terry RL Hunter ZN Yap WT Microparticles bearing encephalitogenic peptides induce T-cell tolerance and ameliorate experimental autoimmune encephalomyelitis. Nat Biotechnol (2012) 30(12):1217–24. 10.1038/nbt.2434 23159881 PMC3589822

[37] Bryant J Hlavaty KA Zhang X Yap WT Zhang L Shea LD Nanoparticle delivery of donor antigens for transplant tolerance in allogeneic islet transplantation. Biomaterials (2014) 35(31):8887–94. 10.1016/j.biomaterials.2014.06.044 25066477 PMC4231141

[38] Maldonado RA LaMothe RA Ferrari JD Zhang AH Rossi RJ Kolte PN Polymeric synthetic nanoparticles for the induction of antigen-specific immunological tolerance. Proc Natl Acad Sci U S A (2015) 112(2):E156–65. 10.1073/pnas.1408686111 25548186 PMC4299193

[39] Yeste A Nadeau M Burns EJ Weiner HL Quintana FJ Nanoparticle-mediated codelivery of myelin antigen and a tolerogenic small molecule suppresses experimental autoimmune encephalomyelitis. Proc Natl Acad Sci U S A (2012) 109(28):11270–5. 10.1073/pnas.1120611109 22745170 PMC3396465

[40] Tsai S Shameli A Yamanouchi J Clemente-Casares X Wang J Serra P Reversal of autoimmunity by boosting memory-like autoregulatory T cells. Immunity (2010) 32(4):568–80. 10.1016/j.immuni.2010.03.015 20381385

[41] Balmert SC Little SR. Biomimetic delivery with micro- and nanoparticles. Adv Mater Deerfield (2012) 24(28):3757–78. 10.1002/adma.201200224 22528985 PMC3627374

[42] Hartwell BL Antunez L Sullivan BP Thati S Sestak JO Berkland C. Multivalent nanomaterials: learning from vaccines and progressing to antigen-specific immunotherapies. J Pharm Sci (2015) 104(2):346–61. 10.1002/jps.24273 25447598

[43] Kohane DS. Microparticles and nanoparticles for drug delivery. Biotechnol Bioeng (2007) 96(2):203–9. 10.1002/bit.21301 17191251

[44] Champion JA Walker A Mitragotri S. Role of particle size in phagocytosis of polymeric microspheres. Pharm Res (2008) 25(8):1815–21. 10.1007/s11095-008-9562-y 18373181 PMC2793372

[45] Isakov N Segal S Immunogenicity of the mutated H-2Kbm1 antigen(s). Test of thyroid graft rejection between B6.C-H-2bm1 and C57BL/6 mice following reciprocal immunization with normal versus malignant cells. Immunobiology (1983) 165(5):485–99. 10.1016/S0171-2985(83)80071-0 6363278

[46] Wang H Wu X Wang Y Oldenborg PA Yang YG. CD47 is required for suppression of allograft rejection by donor-specific transfusion. J Immunol (2010) 184(7):3401–7. 10.4049/jimmunol.0901550 20208011 PMC3670954

[47] Wang W Fang K Wang X Li M Wu Y Chen F Antigen-specific killer polylactic-co-glycolic acid (PLGA) microspheres can prolong alloskin graft survival in a murine model. Immunol Invest (2015) 44(4):385–99. 10.3109/08820139.2015.1014098 25942349

