# Supplementary material for: An Antigen-Presenting and Apoptosis-Inducing Polymer Microparticle Prolongs Alloskin Graft Survival by Selectively and Markedly Depleting Alloreactive CD8+ T Cells
Source: Front Immunol. 2017 Jun 9;8:657. doi: 10.3389/fimmu.2017.00657 (PMC5465244; doi:10.3389/fimmu.2017.00657)
Supplement: Supplementary file 1 [file image_1.pdf]

Supplementary Figure 1:

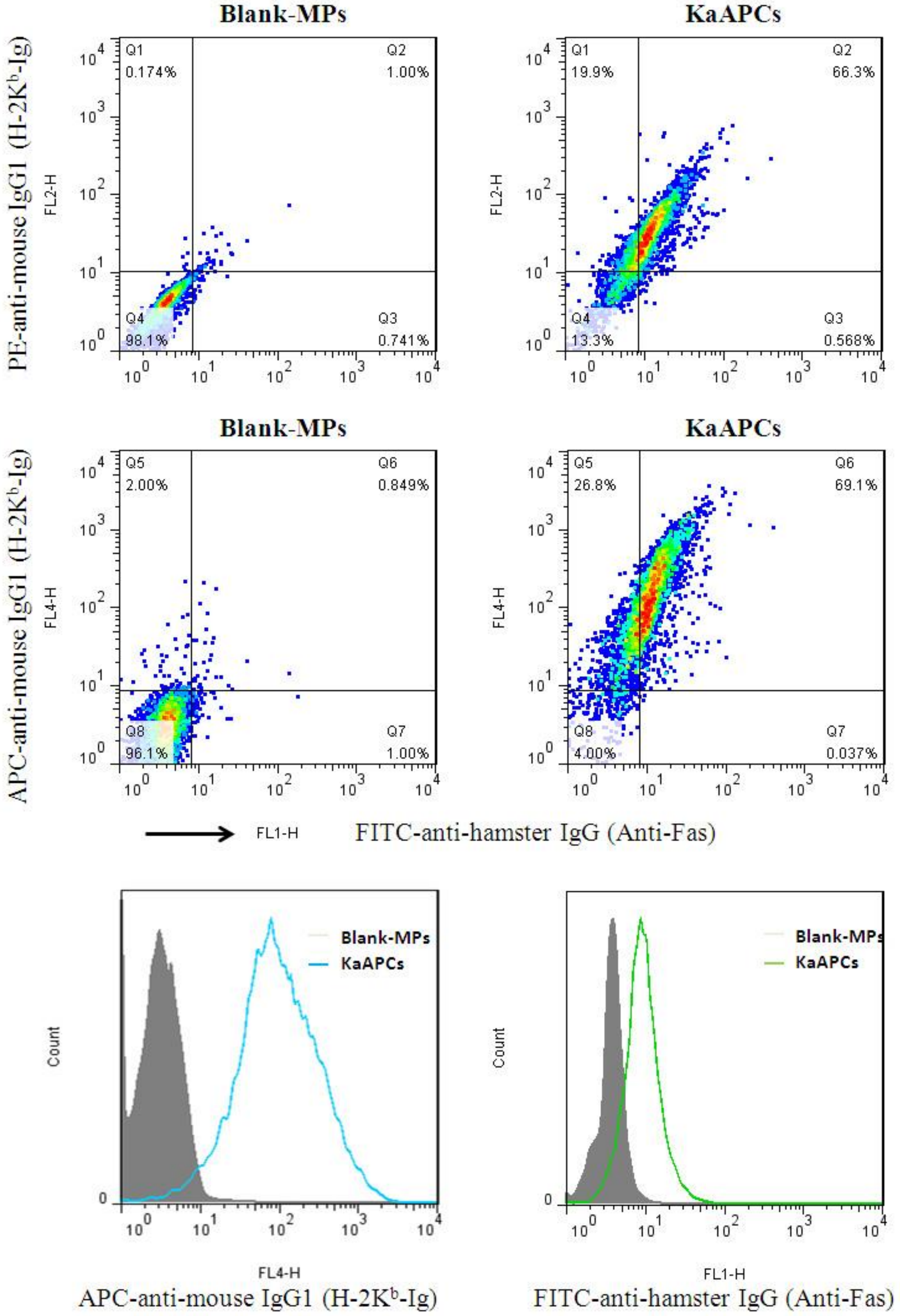

Fig. S1 Phenotypic analyses of KaAPCs. KaAPCs and Blank-MPs (PLGA-MPs blocked with BSA) were stained by PE- or APC-anti-mouse IgG1 (binds to the Ig fragment of H-2K^b-Ig dimer) and FITC-anti-hamster IgG (binds to anti-Fas) mAbs and followed by flow cytometry. The representative dot plots or diagrams were presented.
