# Supplementary material for: An Antigen-Presenting and Apoptosis-Inducing Polymer Microparticle Prolongs Alloskin Graft Survival by Selectively and Markedly Depleting Alloreactive CD8+ T Cells
Source: Front Immunol. 2017 Jun 9;8:657. doi: 10.3389/fimmu.2017.00657 (PMC5465244; doi:10.3389/fimmu.2017.00657)
Supplement: Supplementary file 2 [file image_2.pdf]

Supplementary Figure 2:

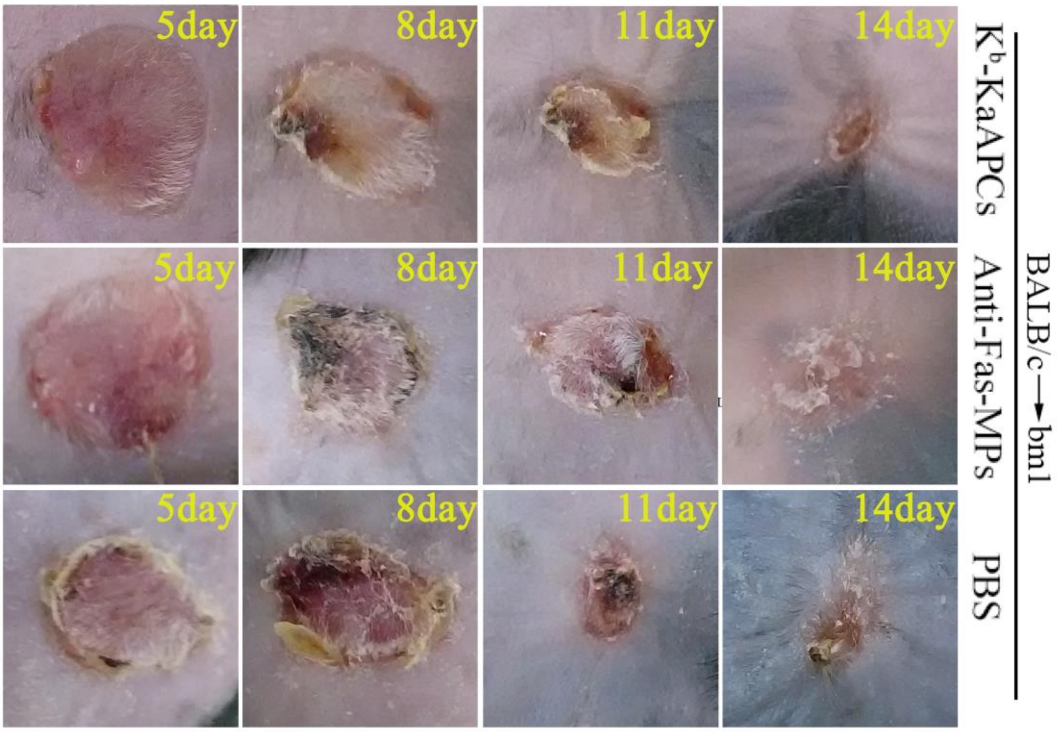

Fig. S2 Representative pictures of alloskin grafts on the indicated days in each treatment group in the third-party transplant model. Ear dorsal tissues of BALB/c mice were grafted onto the dorsal of bm1 mice (a fully MHC-mismatched model), and followed by *i.v.* injection of K^b-KaAPCs, Anti-Fas-MPs (1×10^7 MPs/mouse/time point) or PBS on days 5, 7, and 9 post transplantation. Kaplan-Meier survival plots for alloskin grafts in each treatment group were presented in Fig. 2B.
