# Supplementary material for: An Antigen-Presenting and Apoptosis-Inducing Polymer Microparticle Prolongs Alloskin Graft Survival by Selectively and Markedly Depleting Alloreactive CD8+ T Cells
Source: Front Immunol. 2017 Jun 9;8:657. doi: 10.3389/fimmu.2017.00657 (PMC5465244; doi:10.3389/fimmu.2017.00657)
Supplement: Supplementary file 3 [file image_3.pdf]

Supplementary Figure 3:

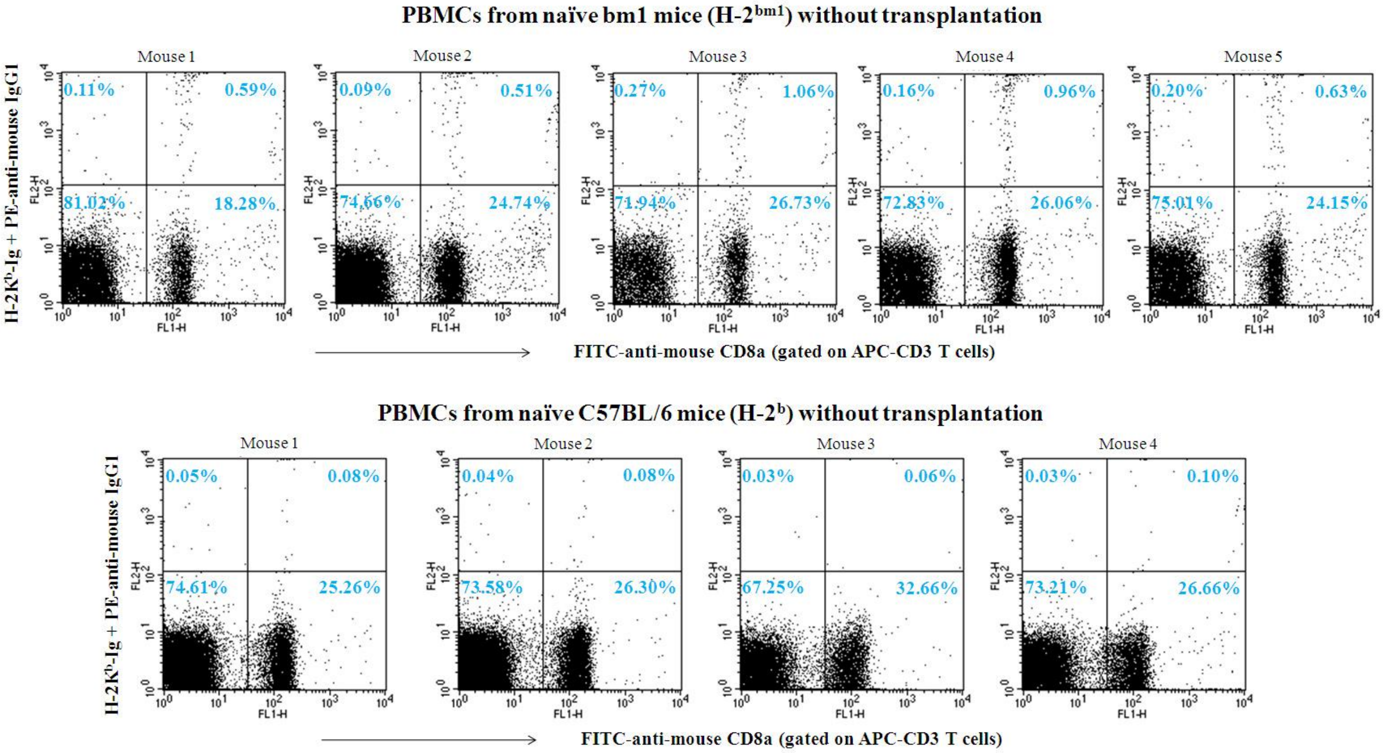

Fig. S3 Percentage of H-2K^b-alloreactive CD8$^+$ T cells and CD4$^+$ T cells in the PBMCs of naïve bm1 mice without transplantation. Before transplantation, the PBMCs of naïve bm1 mice were harvested and stained with H-2K^b-Ig dimer (peptide unloaded), APC-anti-mouse CD3 and FITC-anti-mouse CD8a to detect the frequency of H-2K^b-alloreactive T cells, and followed by flow cytometric analysis of Dimer$^+$/CD8$^+$/CD3$^+$ cells and Dimer$^+$/CD8$^-$/CD3$^+$ cells. As shown in the dot plots, H-2K^b-Ig dimer mainly stained with CD8$^+$ T cells with a percentage of 0.75 ± 0.24% in CD3$^+$ T cell population or 3.01 ± 0.73% in CD8$^+$ T cell population, and very weak staining with CD4$^+$ T cells (CD8$^-$/CD3$^+$) was found (0.16 ± 0.07% in CD3$^+$ T cell population). As a negative control of H-2K^b-Ig dimer staining, PBMCs from naïve C57BL/6 mice (H-2^{b+}) were also stained by H-2K^b-Ig dimer, but only background staining was found with CD8$^+$ T cells.
