# Supplementary material for: An Antigen-Presenting and Apoptosis-Inducing Polymer Microparticle Prolongs Alloskin Graft Survival by Selectively and Markedly Depleting Alloreactive CD8+ T Cells
Source: Front Immunol. 2017 Jun 9;8:657. doi: 10.3389/fimmu.2017.00657 (PMC5465244; doi:10.3389/fimmu.2017.00657)
Supplement: Supplementary file 4 [file image_4.pdf]

Supplementary Figure 4:

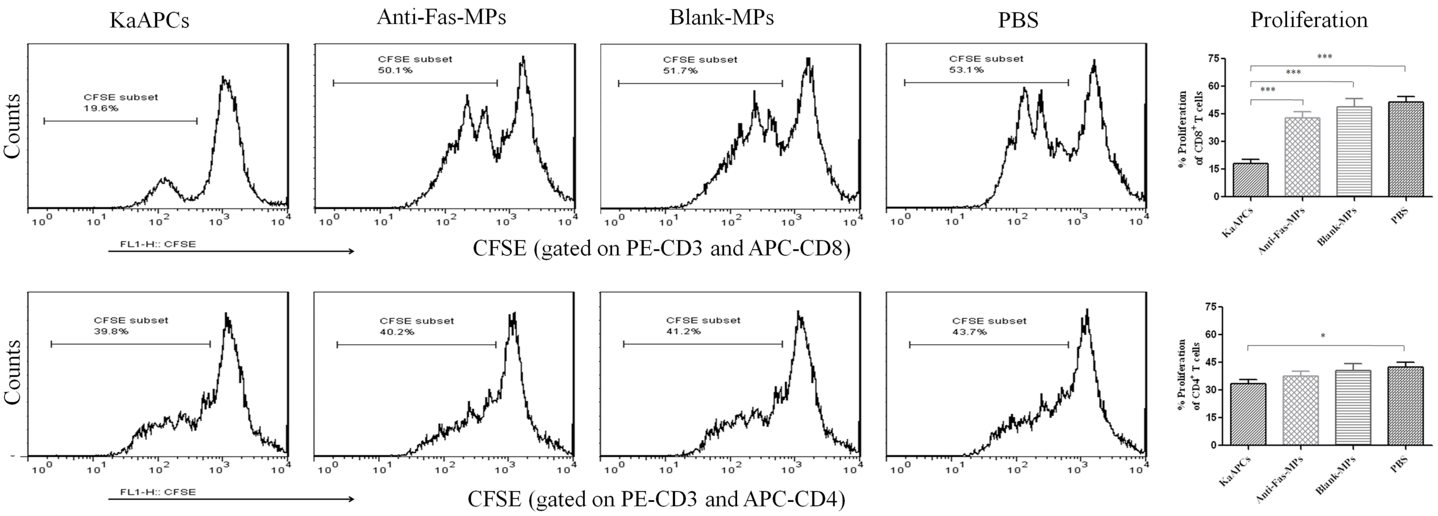

Fig. S4 Allo-proliferation of recipient $CD8^+$ T cells and $CD4^+$ T cells in the anti-donor MLR assays after KaAPCs treatment. Splenocytes from the recipient bm1 mice on day 20 post transplantation in each treatment group were labeled with CFSE and co-cultured with mitomycin C-treated splenocytes from donor C57BL/6 mice for 7 days in 96-well microplates. Then, cells were further stained with PE-anti-mouse CD3 and APC-anti-mouse CD8a (or APC-anti-mouse CD4) mAbs. Proliferation percentage of recipient $CD8^+$ T cells and $CD4^+$ T cells was determined according to cell divisions which were demarcated according to CFSE-staining brightness. The representative histograms of each treatment group were presented. The recipient $CD8^+$ T cells from the KaAPCs group showed nearly 65% reduction of proliferation in response to donor splenocytes, as compared with the recipient $CD8^+$ T cells from the control groups. But the recipient $CD4^+$ T cells from KaAPCs group only displayed a little reduction (21%) of allo-proliferation in the MLR, relative to the PBS control group. *$p<0.05$, **$p<0.01$, and ***$p<0.001$.
