# Supplementary material for: An Antigen-Presenting and Apoptosis-Inducing Polymer Microparticle Prolongs Alloskin Graft Survival by Selectively and Markedly Depleting Alloreactive CD8+ T Cells
Source: Front Immunol. 2017 Jun 9;8:657. doi: 10.3389/fimmu.2017.00657 (PMC5465244; doi:10.3389/fimmu.2017.00657)
Supplement: Supplementary file 5 [file image_5.pdf]

Supplementary Figure 5:

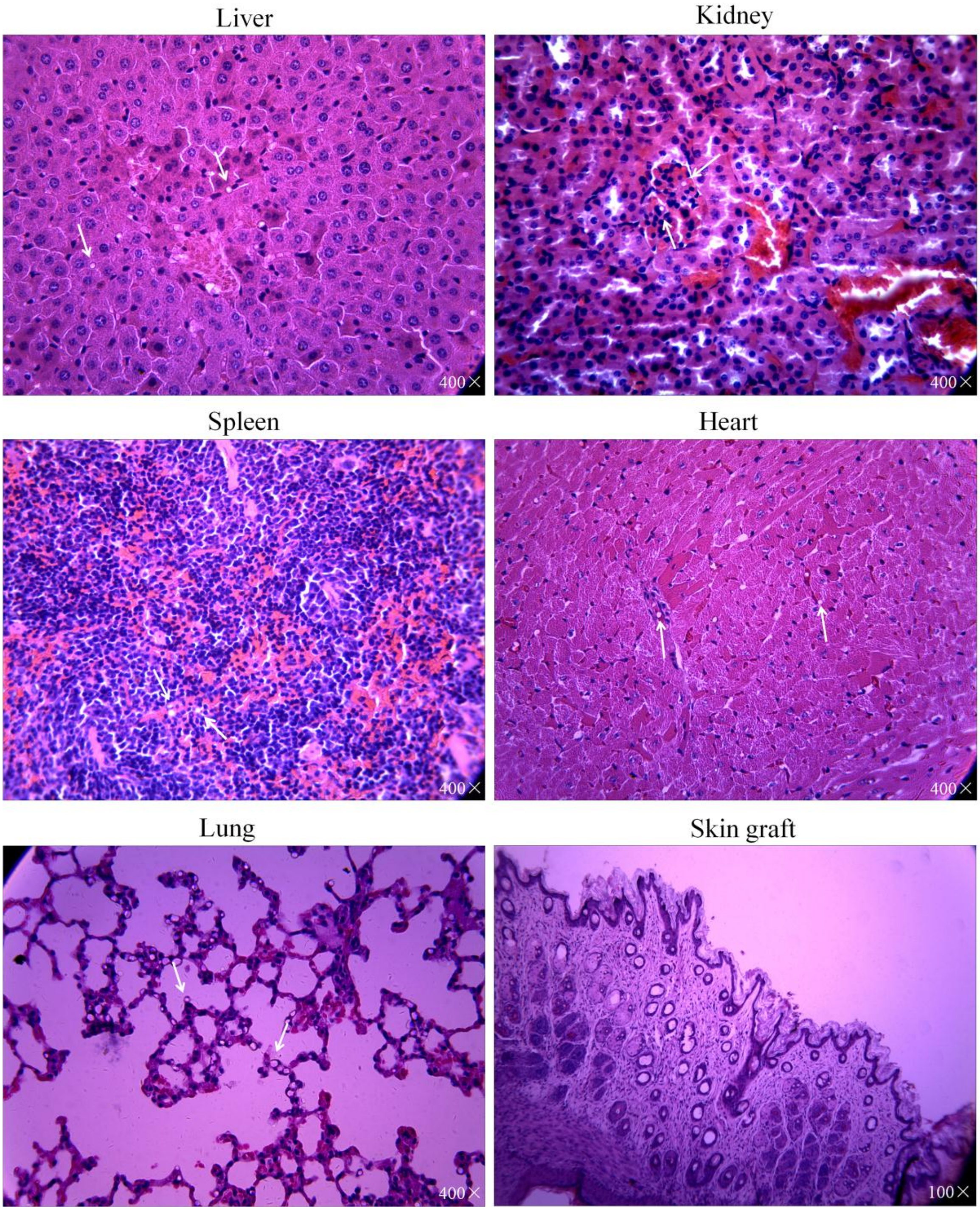

Fig. S5 Presence of KaAPCs in liver, kidney, spleen, lung, and heart, but not skin graft. The KaAPCs were injected via the tail vein into the grafted bm1 mice on day 9 after skin transplantation. At 1 hr after injection, various organs and the full-thickness alloskin graft were dissected and sections were prepared with the thickness of 6μm followed by H&E staining. Finally, the KaAPCs were visualized under optical microscope.
