# Supplementary material for: An Antigen-Presenting and Apoptosis-Inducing Polymer Microparticle Prolongs Alloskin Graft Survival by Selectively and Markedly Depleting Alloreactive CD8+ T Cells
Source: Front Immunol. 2017 Jun 9;8:657. doi: 10.3389/fimmu.2017.00657 (PMC5465244; doi:10.3389/fimmu.2017.00657)
Supplement: Supplementary file 6 [file image_6.pdf]

**Supplementary Figure 6:**

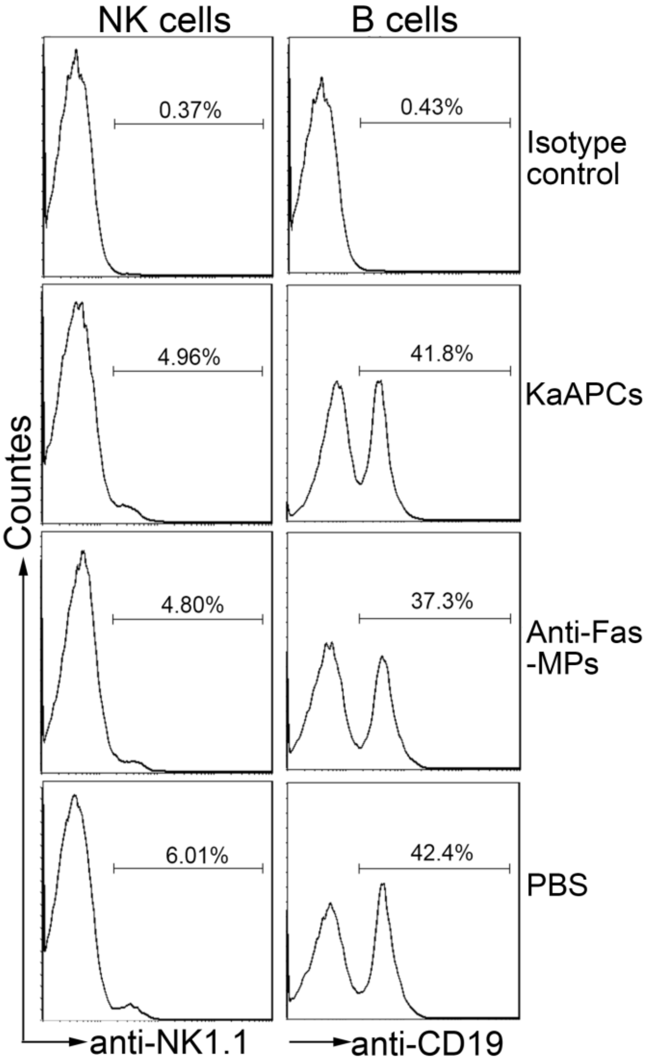


**Fig. S6** Representative diagrams of B cells and NK cells detection. After treatment with KaAPCs, anti-Fas-MPs or PBS on days 9, 11 and 13 after transplantation, splenocytes were prepared from recipient mice on day 15, stained with FITC-anti-mouse CD19 (MB19-1), FITC-anti-mouse NK1.1 (PK136) or isotype control mAbs, and followed by flow cytometry. The frequencies of NK cells and B cells in the gated splenocyte populations were displayed.
