# Supplementary material for: An Antigen-Presenting and Apoptosis-Inducing Polymer Microparticle Prolongs Alloskin Graft Survival by Selectively and Markedly Depleting Alloreactive CD8+ T Cells
Source: Front Immunol. 2017 Jun 9;8:657. doi: 10.3389/fimmu.2017.00657 (PMC5465244; doi:10.3389/fimmu.2017.00657)
Supplement: Supplementary file 7 [file image_7.pdf]

Supplementary Figure 7:

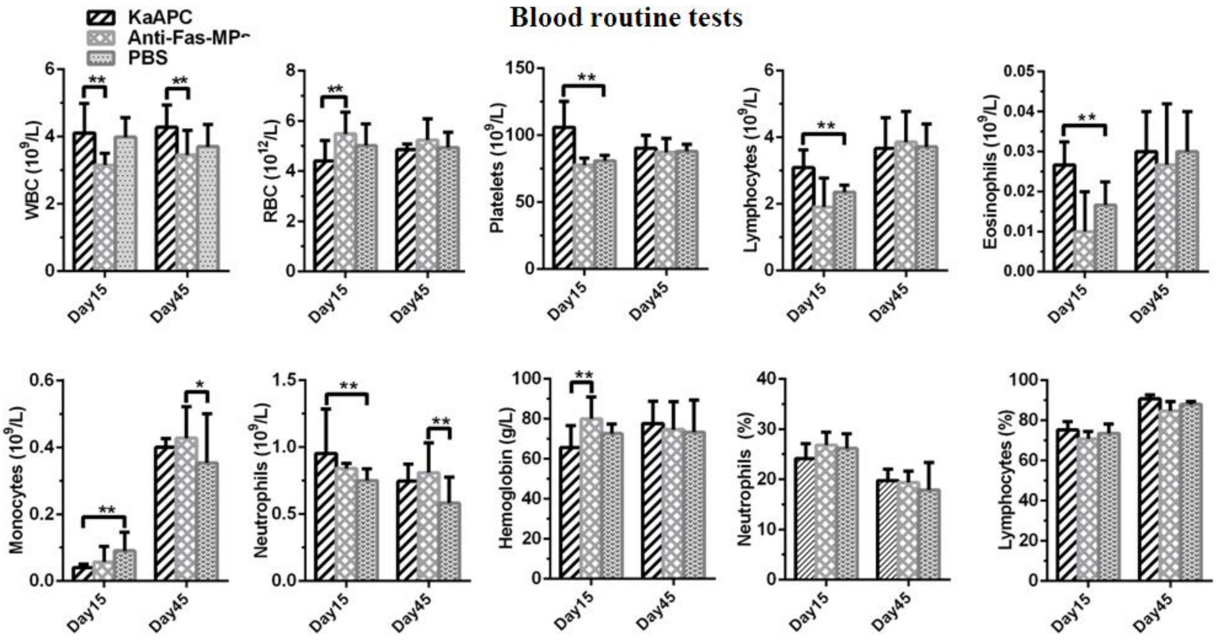

Fig. S7 Blood routine tests at long time point. After treatment with KaAPCs, anti-Fas-MPs or PBS as described, peripheral blood was collected from recipient mice on days 15 and 45 after transplantation (2 days and 32 days after the final treatment) and followed by blood routine tests. Infusions of KaAPCs did not reduce white blood cells (WBC), platelets, lymphocytes, eosinophils, neutrophils, and hemoglobin at two time points when compared to the PBS group, but decreased the numbers of red blood cells (RBC) and monocytes on day 15 as compared with the PBS control group. n = 3 or 4 mice in each group at each time point.
