# Supplementary material for: An Antigen-Presenting and Apoptosis-Inducing Polymer Microparticle Prolongs Alloskin Graft Survival by Selectively and Markedly Depleting Alloreactive CD8+ T Cells
Source: Front Immunol. 2017 Jun 9;8:657. doi: 10.3389/fimmu.2017.00657 (PMC5465244; doi:10.3389/fimmu.2017.00657)
Supplement: Supplementary file 8 [file image_8.pdf]

Supplementary Figure 8:

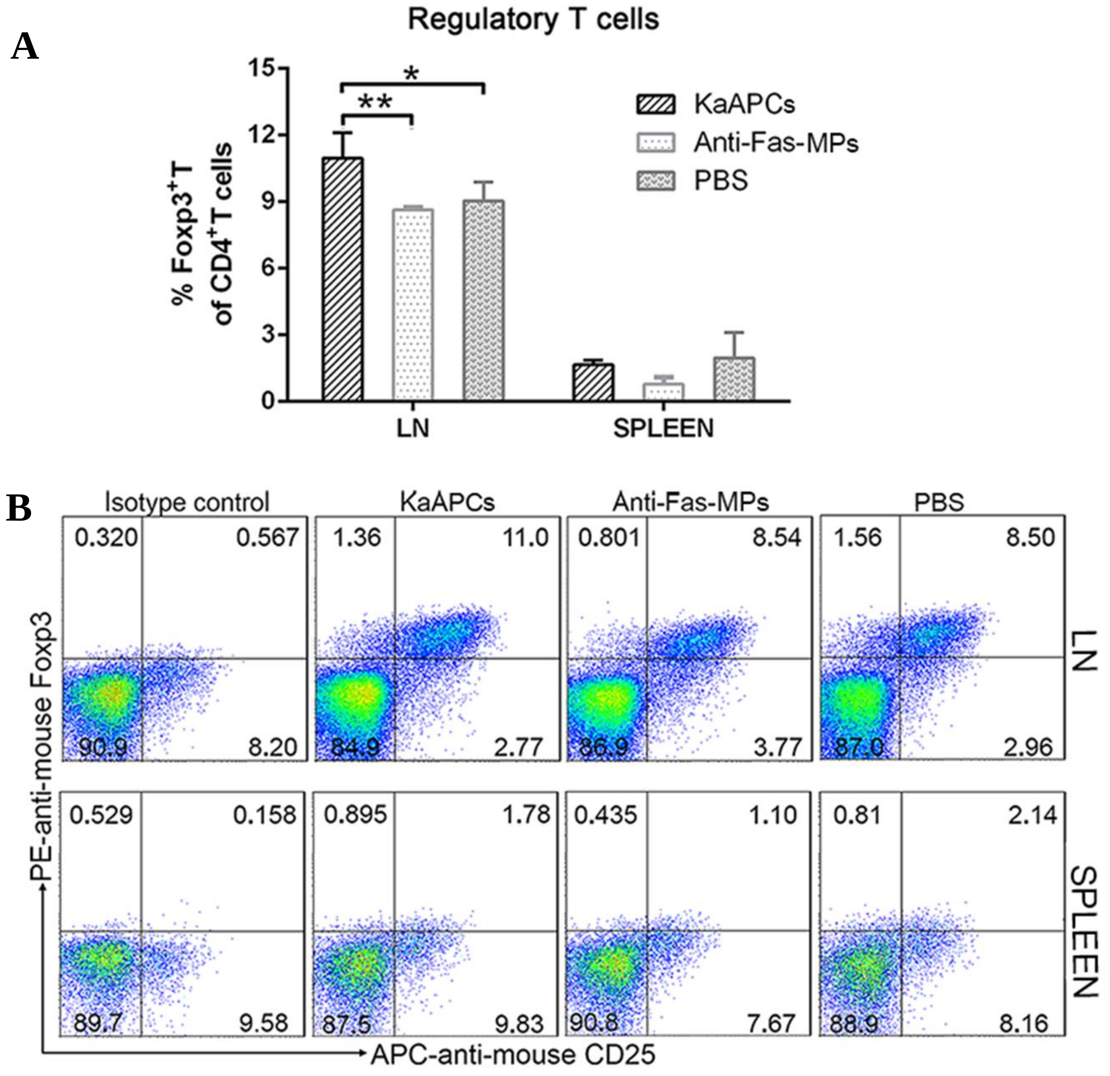

Fig. S8 KaAPCs induce regulatory T cells in lymph nodes. After treatment with KaAPCs, anti-Fas-MPs or PBS on days 9, 11 and 13 after transplantation, splenocytes and lymph node cells were harvested from the recipient mice on day 20. $CD4^+/CD25^+/Foxp3^+$ regulatory T cells (Treg) were detected by flow cytometry. Injections of KaAPCs induced a significantly higher Treg frequency over the control groups in LNs, but not in spleen, as calculated by unpaired, two-tailed Student t-test (A). n = 4 to 6 mice for each group. *p<0.05, **p<0.01. (B) Representative dot plots of Treg detection. Cells were gated on $CD4^+$ T cell population.
