# Supplementary material for: An Antigen-Presenting and Apoptosis-Inducing Polymer Microparticle Prolongs Alloskin Graft Survival by Selectively and Markedly Depleting Alloreactive CD8+ T Cells
Source: Front Immunol. 2017 Jun 9;8:657. doi: 10.3389/fimmu.2017.00657 (PMC5465244; doi:10.3389/fimmu.2017.00657)
Supplement: Supplementary file 9 [file image_9.pdf]

Supplementary Figure 9:

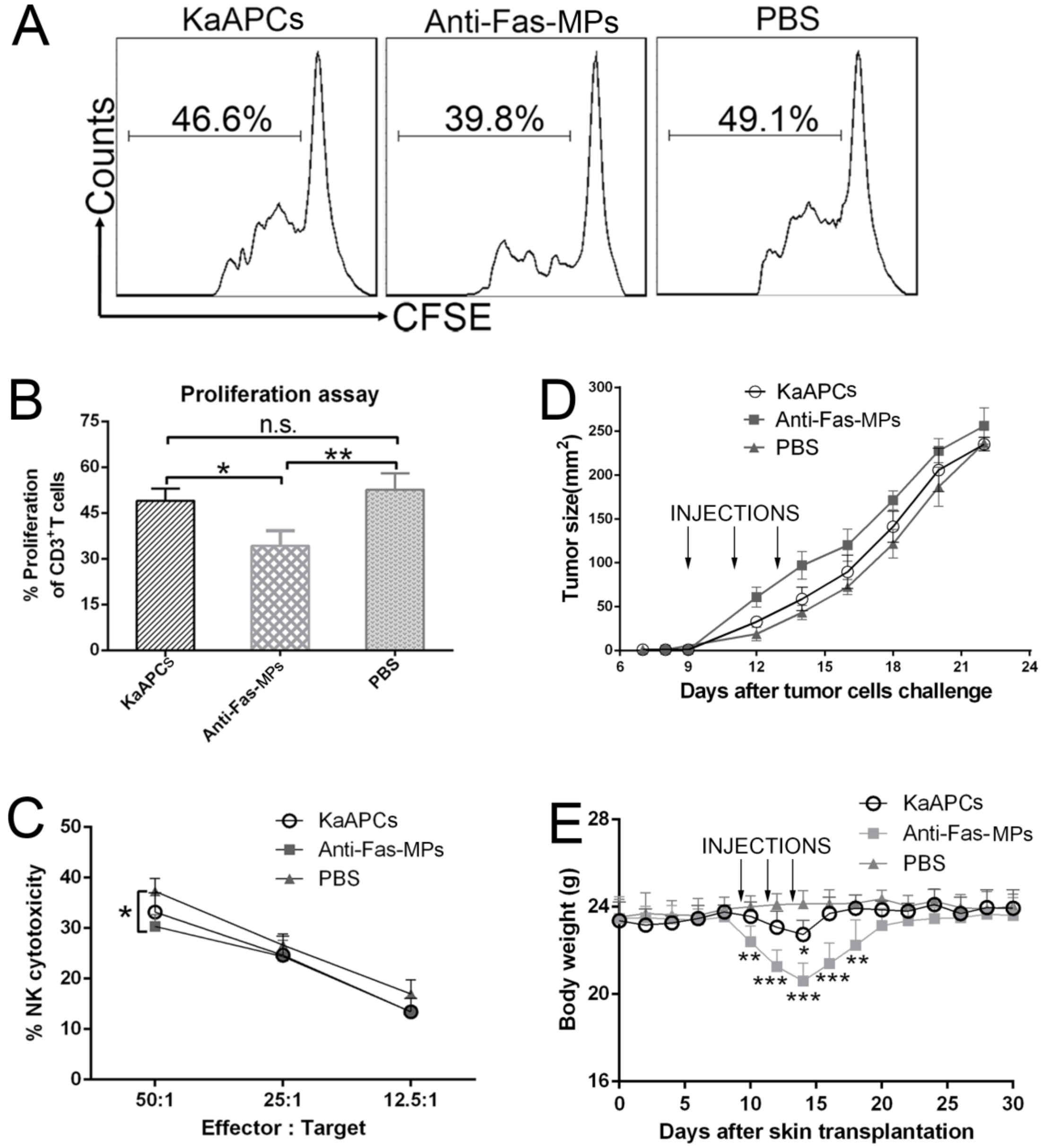

Fig. S9 KaAPCs do not suppress the general immune function of host. After treatment with KaAPCs, anti-Fas-MPs or PBS as described, splenocytes were prepared from recipients on day 15 after transplantation. (A) Representative diagrams of recipient T cell divisions in a third-party MLR. Splenocytes from recipient bm1 mice were labeled with CFSE, co-cultured with the splenocytes of naïve BALB/c mice for 7 days, and followed by APC-anti-CD3e staining and flow cytometry. (B) KaAPCs treatment did not decrease the

proliferation level of recipient T cells in response to the third-party alloantigen. n = 4 to 6 mice in each group. (C) KaAPCs did not impair the cytolysis of recipient NK cells against Yac-1 cells in a 4-hr cytotoxicity assay. n = 4 to 6 mice in each group. Furthermore, grafted bm1 mice were inoculated with B16F10 melanoma cells on day 3 post transplantation and followed by treatment as described. Tumor size (D) and body weight (E) were monitored daily during 22 days after challenge. KaAPCs treatment did not impair host antitumor responses as calculated by Wilcoxon signed rank test, and only led to a transient decrease of body weight after each injection, whereas a discernable weight loss was observed in the Anti-Fas-MPs group, as analyzed by unpaired, two-tailed Student *t* test. n = 10 mice for each group. Arrows point at the time points of KaAPCs, anti-Fas-MPs or PBS injection. *p<0.05, **p<0.01, and ***p<0.001.
