# Supplementary material for: An Antigen-Presenting and Apoptosis-Inducing Polymer Microparticle Prolongs Alloskin Graft Survival by Selectively and Markedly Depleting Alloreactive CD8+ T Cells
Source: Front Immunol. 2017 Jun 9;8:657. doi: 10.3389/fimmu.2017.00657 (PMC5465244; doi:10.3389/fimmu.2017.00657)
Supplement: Supplementary file 10 [file image_10.pdf]

Supplementary Figure 10:

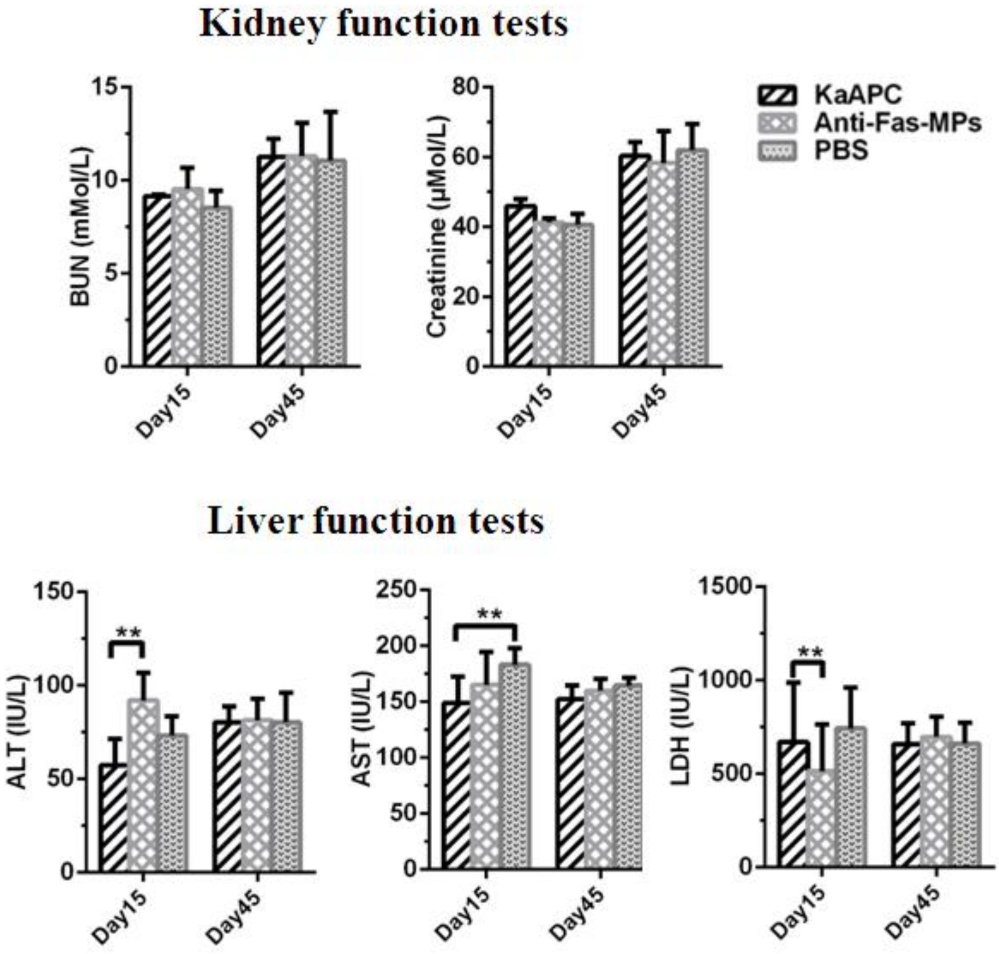

Fig. S10 Functional evaluation of liver and kidney at long time point. After treatment with KaAPCs, anti-Fas-MPs or PBS as described, peripheral blood was collected from recipient mice on days 15 and 45 after transplantation (2 days and 32 days after the final treatment) and followed by biochemical routine tests for the functional evaluation of liver and kidney. Infusions of KaAPCs did not statistically increase the concentrations of biochemical parameters in serum as compared with the PBS group. BUN: blood urea nitrogen; AST: aspartate aminotransferase; ALT: alanine aminotransferase; LDH: lactate dehydrogenase; CR: creatinine. n = 3 or 4 mice in each group at each time point. **$p<0.01$.
