# Supplementary material for: An Antigen-Presenting and Apoptosis-Inducing Polymer Microparticle Prolongs Alloskin Graft Survival by Selectively and Markedly Depleting Alloreactive CD8+ T Cells
Source: Front Immunol. 2017 Jun 9;8:657. doi: 10.3389/fimmu.2017.00657 (PMC5465244; doi:10.3389/fimmu.2017.00657)
Supplement: Supplementary file 11 [file image_11.pdf]

Supplementary Figure 11:

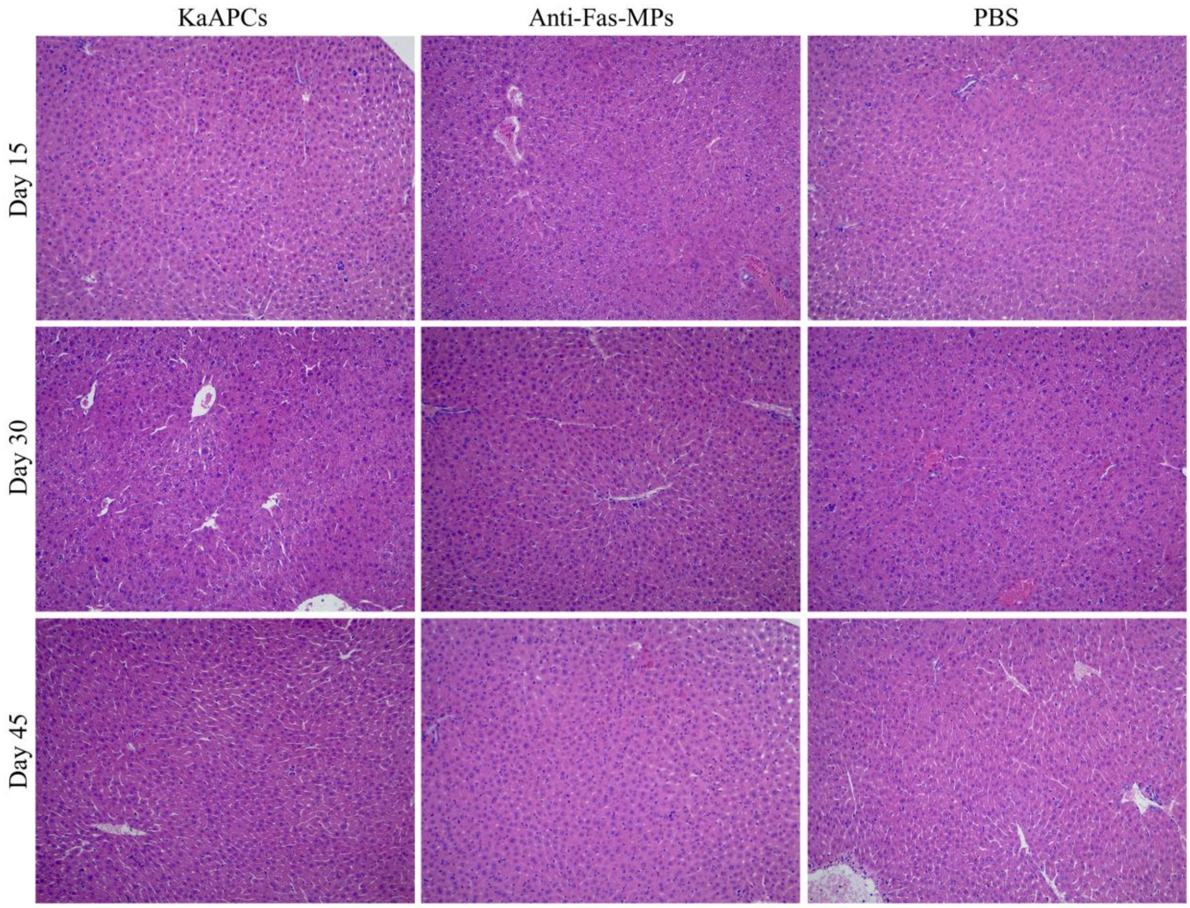

Fig. S11 KaAPCs do not cause visible pathological injuries in liver. After treatment with KaAPCs, anti-Fas-MPs or PBS as described, the pathological injuries were analyzed by H&E staining for various organs on days 15, 30, and 45 after transplantation (2 days, 17 days, and 32 days after the final treatment). Representative staining results of liver sections from each treatment group at each time point were presented. n = 3 mice for each group at each time point.
