# Supplementary material for: An Antigen-Presenting and Apoptosis-Inducing Polymer Microparticle Prolongs Alloskin Graft Survival by Selectively and Markedly Depleting Alloreactive CD8+ T Cells
Source: Front Immunol. 2017 Jun 9;8:657. doi: 10.3389/fimmu.2017.00657 (PMC5465244; doi:10.3389/fimmu.2017.00657)
Supplement: Supplementary file 13 [file image_13.pdf]

Supplementary Figure 13:

A **Kidney sections**

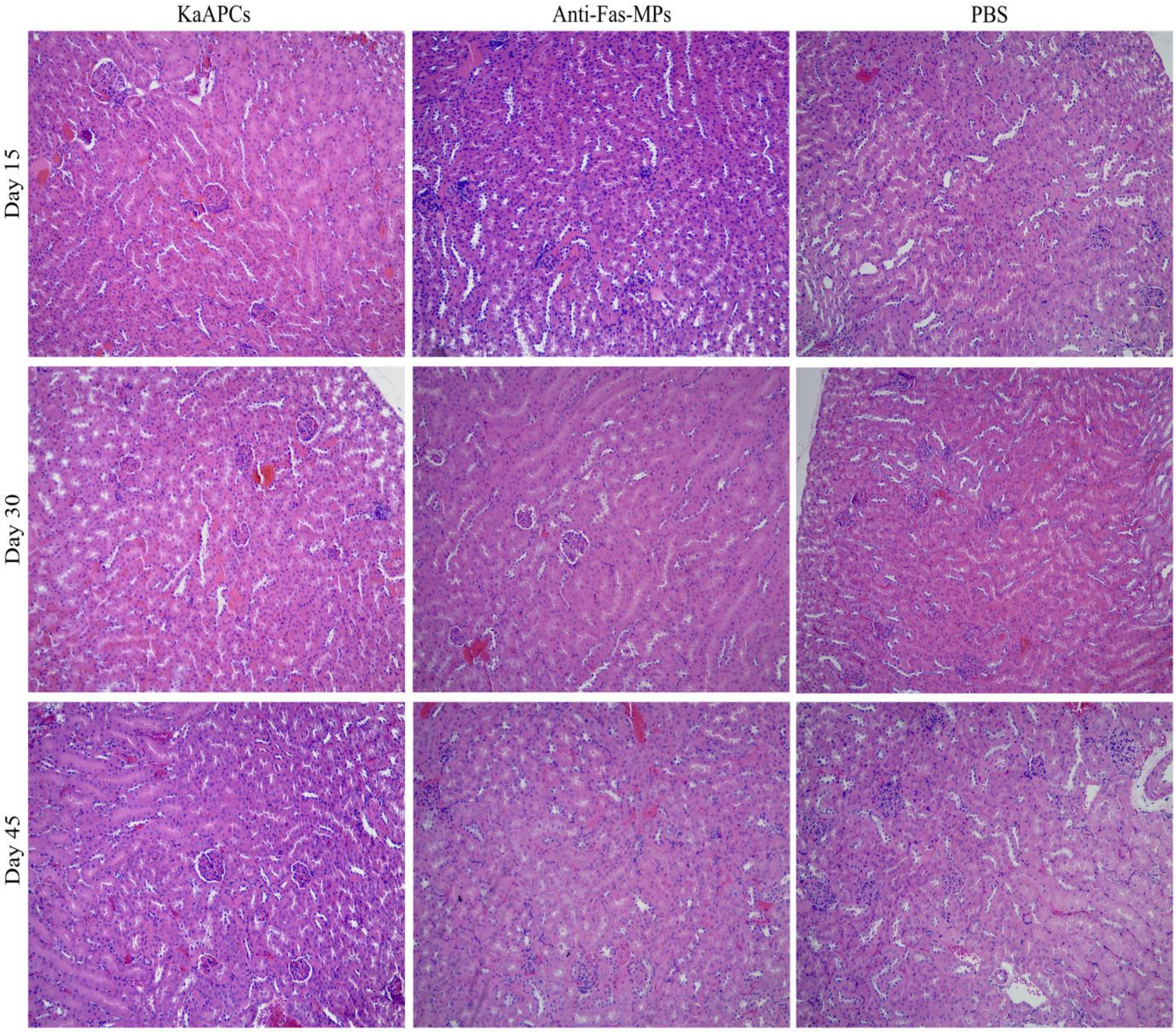

B **Heart sections**

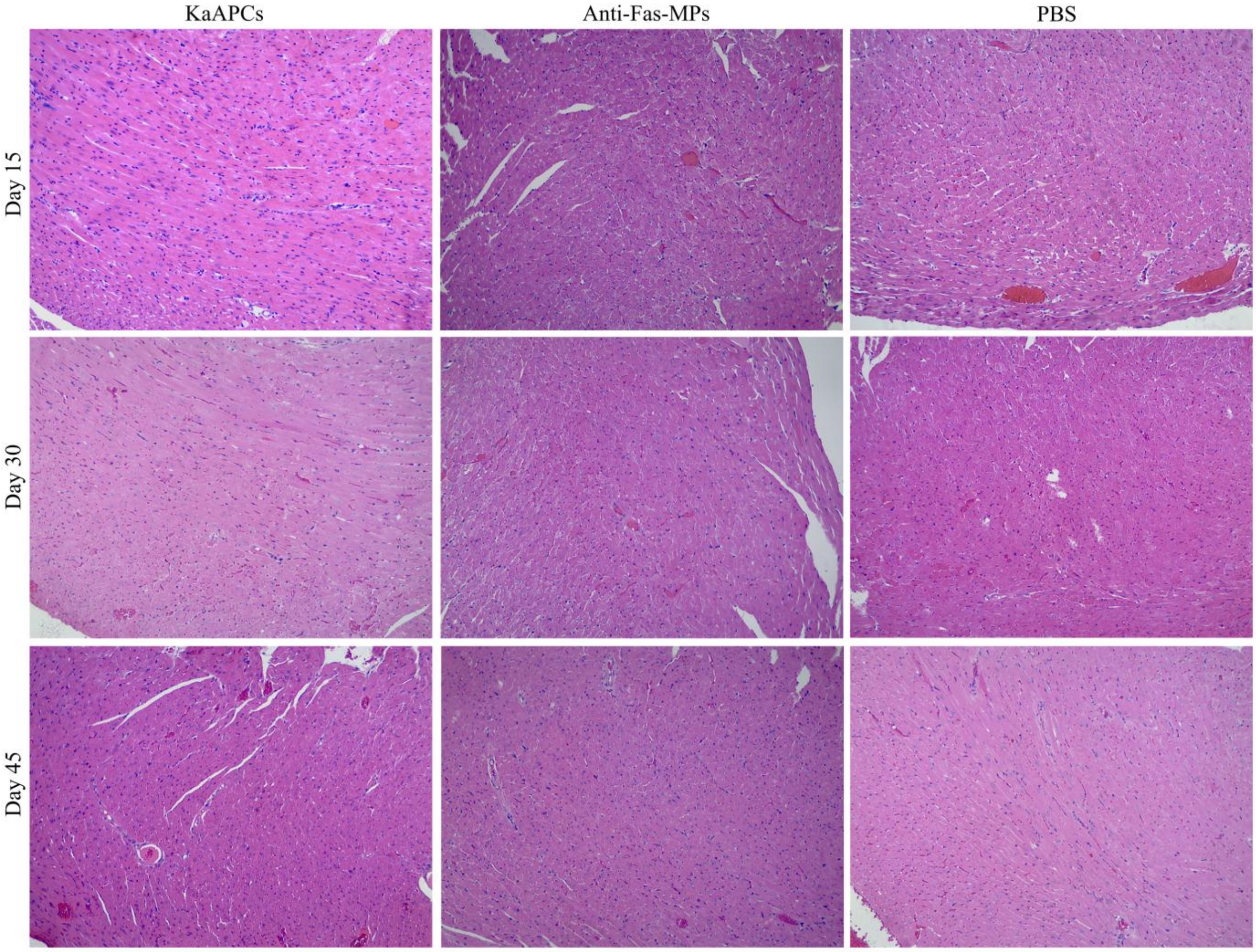

C Lung sections

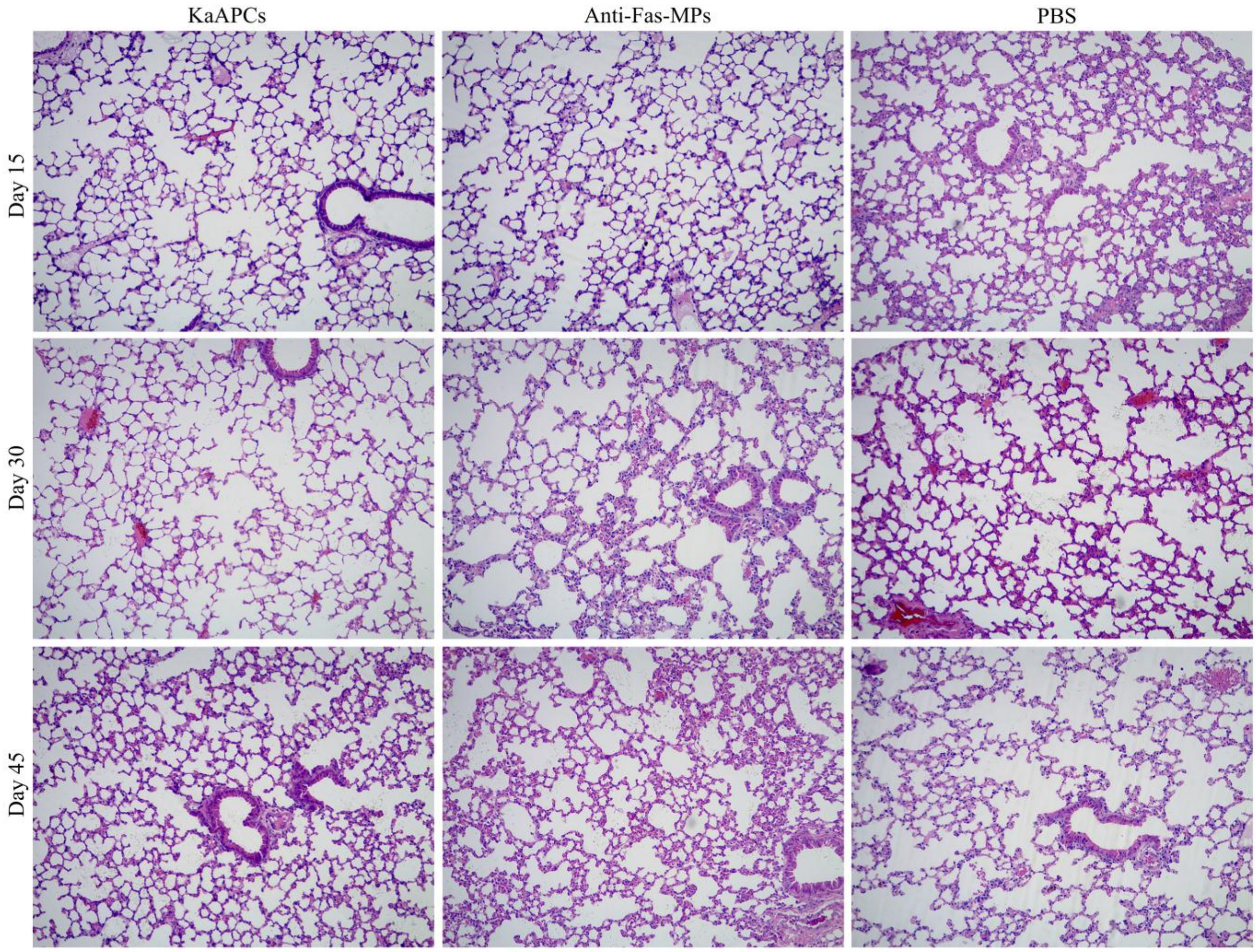

Fig. S13 KaAPCs do not cause visible pathological injuries in kidney, heart, and lung. After treatment with KaAPCs, anti-Fas-MPs or PBS as described, the pathological injuries were analyzed for various organs on days 15, 30, and 45 after transplantation by H&E staining. Representative staining results of kidney sections (A), heart sections (B), and lung sections (C) from each treatment group at each time point were presented. n = 3 mice for each group at each time point.
